# Elevated cerebrospinal fluid cytokine levels in tuberculous meningitis predict survival in response to dexamethasone

**DOI:** 10.1073/pnas.2024852118

**Published:** 2021-03-03

**Authors:** Laura J. Whitworth, Rajan Troll, Antonio J. Pagán, Francisco J. Roca, Paul H. Edelstein, Mark Troll, David M. Tobin, Nguyen Hoan Phu, Nguyen Duc Bang, Guy E. Thwaites, Nguyen Thuy Thuong Thuong, Roger F. Sewell, Lalita Ramakrishnan

**Affiliations:** ^a^Molecular Immunity Unit, Department of Medicine, University of Cambridge, CB2 0QH Cambridge, United Kingdom;; ^b^Medical Research Council Laboratory of Molecular Biology, CB2 0QH Cambridge, United Kingdom;; ^c^Trinity College, CB2 1TQ Cambridge, United Kingdom;; ^d^Department of Pathology and Laboratory Medicine, Perelman School of Medicine, University of Pennsylvania, Philadelphia, PA 19104;; ^e^Department of Molecular Genetics and Microbiology, Duke University School of Medicine, Durham, NC 27710;; ^f^Department of Immunology, Duke University School of Medicine, Durham, NC 27710;; ^g^Oxford University Clinical Research Unit, Ho Chi Minh City, Vietnam;; ^h^Hospital for Tropical Diseases, Ho Chi Minh City, Vietnam;; ^i^Pham Ngoc Thach Hospital for Tuberculosis and Lung Disease, Ho Chi Minh City, Vietnam;; ^j^Centre for Tropical Medicine and Global Health, Nuffield Department of Medicine, University of Oxford, OX3 7BN Oxford, United Kingdom

**Keywords:** tuberculous meningitis, cytokines, inflammation, corticosteroids, Bayesian analysis

## Abstract

Adjunctive treatment with antiinflammatory corticosteroids like dexamethasone increases survival in tuberculosis meningitis. Dexamethasone responsiveness associates with a C/T variant in *Leukotriene A4 Hydrolase* (*LTA4H*), which regulates expression of the proinflammatory mediator leukotriene B_4_ (LTB_4_). TT homozygotes, with increased expression of *LTA4H*, have the highest survival when treated with dexamethasone and the lowest survival without. While the T allele is present in only a minority of the world’s population, corticosteroids confer modest survival benefit worldwide. Using Bayesian methods, we examined how pretreatment levels of cerebrospinal fluid proinflammatory cytokines affect survival in dexamethasone-treated tuberculous meningitis. *LTA4H* TT homozygosity was associated with global cytokine increases, including tumor necrosis factor. Association between higher cytokine levels and survival extended to non-TT patients, suggesting that other genetic variants may also induce dexamethasone-responsive pathological inflammation. These findings warrant studies that tailor dexamethasone therapy to pretreatment cerebrospinal fluid cytokine concentrations, while searching for additional genetic loci shaping the inflammatory milieu.

## Significance

Despite appropriate antibiotic treatment, tuberculous meningitis carries a high mortality ascribed to overexuberant inflammation. Genetic variations in the enzyme, LTA4H, alter inflammation, with individuals carrying the inflammation-associated *LTA4H* variant benefitting from antiinflammatory steroids administered alongside antibiotics. A prior study found poor correlation between *LTA4H* genotype and cerebrospinal fluid levels of cytokines, key mediators of inflammation. The study used “frequentist” statistical methods that can fail to detect true differences. Using Bayesian statistics, which can detect significant differences not found by frequentist methods, we found good correlation between *LTA4H* genotype and cytokine levels, and cytokine levels and outcome even independent of *LTA4H* genotype. These findings suggest that LTA4H and additional inflammation factors affect outcome and suggest tailoring steroid therapy to cytokine levels.

Tuberculous meningitis is the most lethal form of tuberculosis, with a mortality of 25 to 40% in drug-sensitive HIV uninfected adults (1–3). Drug-resistant infection and HIV coinfection leads to even higher mortality (1, 3). Because multiple investigations suggest that dysregulated inflammation plays a role in mortality from this disease, corticosteroids, which are broadly acting antiinflammatory drugs, are now routinely used as adjunctive therapy to antitubercular antibiotics (4–6). The relatively modest reduction of mortality with corticosteroids suggests that tuberculous meningitis may elicit different inflammatory responses, with corticosteroids helping those with high levels of inflammation. Genetic variation is likely to control these heterogeneous responses and a common functional variant in the *Leukotriene A4 Hydrolase* (*LTA4H*) gene is associated with responsiveness to dexamethasone, a potent corticosteroid (7–9). LTA4H is a key enzyme in arachidonic acid metabolism that catalyzes the production of leukotriene B_4_, a proinflammatory lipid mediator with pleiotropic inflammatory effects (10, 11). A C/T transition in the promoter modulates the *LTA4H* gene and, thereby, protein expression. Consistent with its expression mediating an inflammatory milieu, CC and TT homozygotes have the lowest and highest LTA4H expression, respectively, with intermediate expression in CT heterozygotes. TT homozygotes have the greatest survival benefit from dexamethasone while suffering the highest mortality among those not given this drug (8).

The role of LTA4H in controlling inflammation and survival in the context of mycobacterial infections was first identified in a zebrafish forward genetic screen, where animals with both low and high *LTA4H* expression were more susceptible to *Mycobacterium marinum* infection than their wild-type counterparts (8, 12). In the zebrafish, high *LTA4H*-mediated susceptibility is due to its increased product LTB_4_ inducing excessive tumor necrosis factor (TNF) (8). TNF causes pathogenic programmed necrosis of macrophages in *M. marinum-*infected zebrafish as well as *Mycobacterium tuberculosis-*infected human macrophages (13, 14).

Because tuberculous meningitis is characterized by a necrotizing granulomatous reaction and macrophage-rich meningeal exudates (15, 16), we wanted to determine if the *LTA4H* TT genotype mediates increases in cerebrospinal fluid (CSF) cytokines and if these increases are associated with dexamethasone responsiveness. Consistent with the findings of Tobin et al. (8), analysis of a second Vietnam tuberculous meningitis cohort, where all individuals had been treated with dexamethasone, showed that *LTA4H* TT HIV-uninfected individuals had increased survival over their non-TT counterparts (7, 9). The same study also determined CSF cytokine levels to test the prediction that TT individuals have a hyperinflammatory CSF profile reflected by elevated cytokines (7). The original analysis of the cytokine profiles in this study was conducted using linear trend tests and found that the median levels of all 10 assayed cytokines were increased in TT compared to CC and CT patients, but in a multiple-comparison test only the interleukins (IL) IL-1β, IL-2, and IL-6 increases achieved statistical significance (7).

Here, we have reanalyzed the cytokine data from these HIV-uninfected adults with tuberculous meningitis using Bayesian methods. Bayesian analysis can detect significant results and relationships not detected by frequentist methods because they do not impose a penalty for multiple comparisons and can effectively detect significant differences that are hidden by type 2 errors in frequentist analysis (9). Moreover, we don’t know the class of distribution (e.g., normal, Gamma, and so forth) from which the cytokine values come. Bayesian methods can identify the likely class of distribution and take this information into account for the analyses even without having surety about the correct class.

Using Bayesian methods (detailed in *SI Appendix*, Appendices S1 and S2), we find that survival in response to dexamethasone is associated with significant increases in all cytokines tested before or at the start of treatment, representing innate proinflammatory, helper T cell-associated and immunomodulatory classes. While the *LTA4H* TT genotype was associated with increases in these cytokines, we also found that increased cytokines are associated with survival in an *LTA4H*-independent manner in this dexamethasone-treated cohort.

## Results

### In Tuberculous Meningitis Patients, *LTA4H* TT Genotype Is Associated with Increased CSF Levels of Multiple Cytokines, including TNF.

**Fig. 1.**
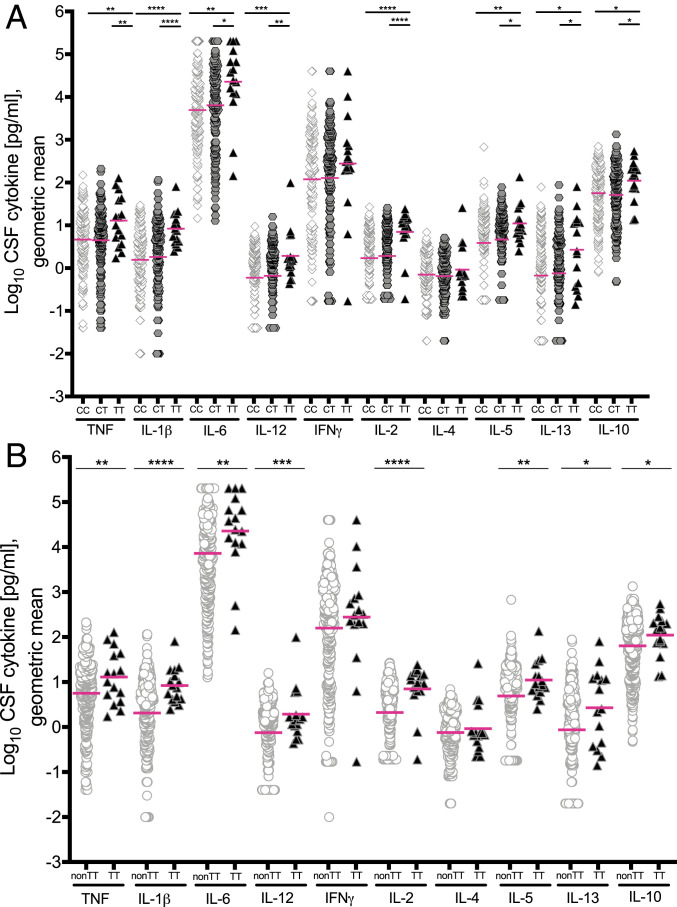
CSF cytokine levels grouped by *LTA4H* genotype. (*A*) Cytokine levels in CSF from CC (*n* = 148), CT (*n* = 142), and TT (*n* = 16) patients. (*B*) Cytokine levels compared between non-TT (*n* = 290) and TT (*n* = 16) genotypes. Magenta lines indicate geometric means. Asterisks indicate probability that right-hand group values are significantly greater than the left (* ≥ 0.95, ** ≥ 0.99, *** ≥ 0.999, **** ≥ 0.9999). Unspecified comparisons are not significant.

Analysis of the CSF cytokine values from Thuong et al. (7) (Dataset S1) showed that none followed a normal (Gaussian) distribution. For nearly all values, log-skew-Student (log-noncentral-*t* distribution) was the preferred distribution class both in the dataset as a whole and for the various subsets considered in our analyses (*SI Appendix*, Appendix S2). Comparisons were performed using restricted geometric means as is appropriate for such heavy-tailed approximately logarithmically distributed data (see definitions of statistical terms used in *SI Appendix*, Supplementary Box S1 and Appendix S2). Furthermore, unlike the previous analysis, we made no assumption that there would be a linear trend with the number of T-alleles in a given patient. Using this method of analysis, we found that TT patients had significant increases in all measured CSF cytokines, except interferon-γ (IFN-γ) and IL-4, compared to both CC and CT patients who had similar levels to one another (Fig. 1*A* and Dataset S2). Similarly, a comparison of cytokine levels in TT patients to those in combined non-TT (CT and CC) patients showed that these levels in TT patients were significantly higher for all cytokines except for IFN-γ and IL-4 (Fig. 1*B*). In both comparisons, IFN-γ and IL-4 were also increased though the differences were not significant. The finding that a single T allele does not have a discernible influence on inflammatory pathways is consistent with the CC and CT patients in this cohort having similarly lower survival than TT patients when all patients were receiving dexamethasone therapy (7, 9). Thus, TT homozygosity is associated with increased cytokine concentrations across the board, including cytokines that are associated with: An acute inflammatory response (TNF, IL-1β, and IL-6), T cell activation and regulatory T cell homeostasis (IL-2), innate and adaptive type-1 immunity (IL-12 and IFN-γ), innate and adaptive type-2 immunity (IL-4, IL-5, and IL-13), and immune modulation (IL-10). Importantly, TNF, which drives the pathogenesis caused by LTA4H excess in the zebrafish model of tuberculosis (8, 13, 14) is significantly increased in TT patients.

### The *LTA4H *TT Genotype Exerts a Compensatory Regulation on CSF Cytokine Levels in More Severe Disease.

**Fig. 2.**
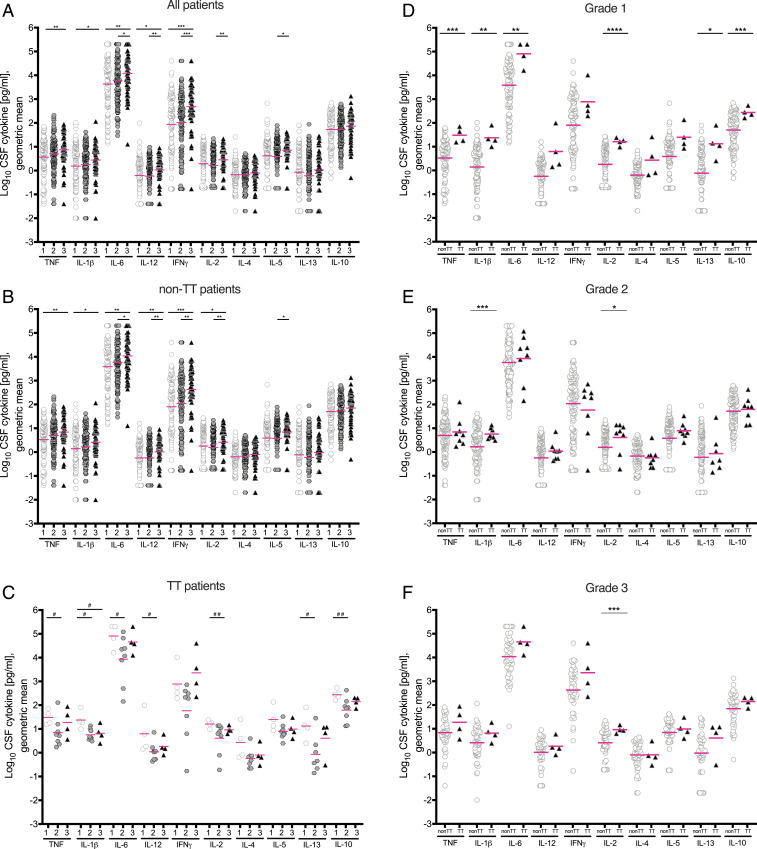
Cytokine levels by grade and by genotype. (*A*) All patients (grade 1 *n* = 109, grade 2 *n* = 141, grade 3 *n* = 55); (*B*) Non-TT patients (grade 1 *n* = 105, grade 2 *n* = 133, grade 3 *n* = 51), and (*C*) TT patients (grade 1 *n* = 4, grade 2 *n* = 8, grade 3 *n* = 4). (*D*) Grade 1 patients; (*E*) grade 2 patients; (*F*) grade 3 patients. Magenta lines indicate geometric means. Asterisks indicate probability that right-hand group values are significantly greater than the left (* ≥ 0.95, ** ≥ 0.99, *** ≥ 0.999, **** ≥ 0.9999). Hash symbols indicate probability that left-hand group values are significantly greater than the right (^#^ ≥0.95, ^##^ ≥0.99). Unspecified comparisons are not significant.

Tuberculous meningitis patients can present with a wide-ranging disease severity, reflected by the presence or absence of focal neurological signs, or a generalized decrease in responsiveness including coma (17). The modified British Medical Research Council (BMRC) tuberculous meningitis grading system categorizes patients into three grades in increasing order of severity (3, 17). Prior analysis of this cohort found that disease grade was associated with a trend to increased cytokines across the board with a significant increase for only one, IFN-γ (7). Our analysis found all to be increased with increasing grade, with significant increases between grades for 7 of the 10 (Fig. 2*A* and Dataset S2). Since there were increased cytokine levels for both the TT genotype and for higher disease grades, we predicted that these levels would be highest in TT patients in the higher disease grades. Whereas non-TT patients had a similar pattern of increased cytokines with increasing disease grade as the overall cohort (Fig. 2*B*), we were surprised to find that in TT patients, the pattern was reversed. The majority of the cytokines were lower in grades 2 and 3 than in grade 1, significantly so in many cases (Fig. 2*C*). The major shift occurred between grades 1 and 2. Grade 3 cytokines were not lower than grade 2; the levels were either similar in these two grades or grade 2 levels were nonsignificantly lower. These findings suggest the existence of compensatory mechanisms in TT patients that limit extreme increases in cytokine levels driven by increased disease severity. Consistent with this hypothesis, when we compared cytokine levels in non-TT to those in TT patients stratified by disease grade, cytokine levels in TT patients were higher in all grades, with increases that were the greatest and most significant in grade 1, rather than in grades 2 and 3 (Fig. 2 *D*–*F*).

### Both *LTA4H *TT-Dependent and -Independent CSF Cytokine Increases Are Associated with Survival in Response to Dexamethasone.

**Fig. 3.**
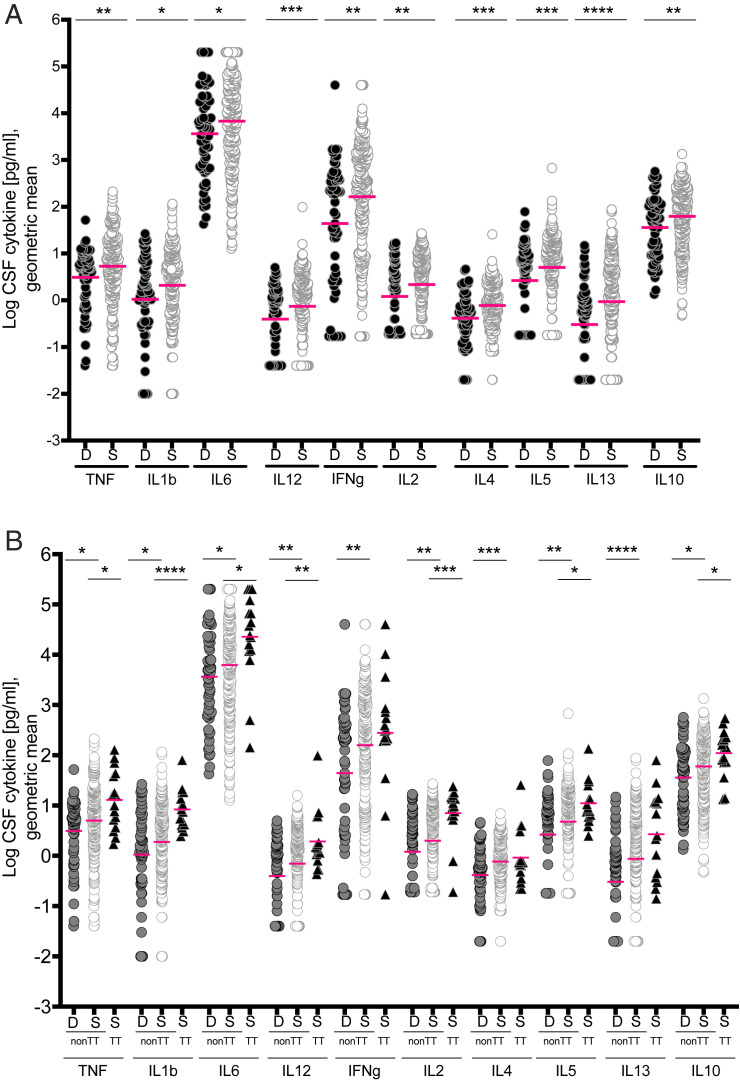
Cytokine levels in survivors and nonsurvivors. (*A*) Cytokine levels in patients who survived (S, *n* = 248) versus those who died (D, *n* = 57); (*B*) separated by genotypes into non-TT (deaths, *n* = 57, survivors, *n* = 232) and TT (all survived, *n* = 16). Magenta lines indicate geometric means. Asterisks indicate probability that right-hand group values are significantly greater than the left (* ≥ 0.95, ** ≥ 0.99, *** ≥ 0.999, **** ≥ 0.9999). Comparisons performed for each cytokine: Non-TT dead vs. survived and non-TT survived vs. TT survived. Cytokine levels between non-TT dead and TT survived were not compared.

Thuong et al. (7) compared cytokine levels independent of *LTA4H* genotype in tuberculous meningitis survivors to nonsurvivors following adjunctive dexamethasone treatment and found that survivors had increased cytokine levels. Our reanalysis confirmed this result; all cytokines were significantly increased in survivors compared to those who died (Fig. 3*A*). Because TT patients had significantly increased survival with dexamethasone as compared to non-TT patients (7, 9), we hypothesized that the increased cytokine levels in survivors overall would be restricted to TT patients. However, even among non-TT patients only, survivors had significantly increased cytokines across the board when compared to those who died (Fig. 3*B*). We could not compare TT survivors to nonsurvivors as the four TT patients who died did not have CSF cytokine measurements. Comparison of TT survivors to non-TT survivors revealed that most cytokines were significantly higher in TT survivors than in the non-TT survivors (Fig. 3*B*). CC and CT survivors each also had higher cytokines than nonsurvivors overall and in all three disease grades (*SI Appendix*, Fig. S1). Together, these analyses show that the default inflammatory response to tuberculous meningitis includes global increases in CSF cytokines, and suggest they are associated with a survival benefit from dexamethasone. Overlaid on these are further increases mediated by the *LTA4H* TT genotype.

The finding that the *LTA4H* TT patients have increased CSF cytokines over their non-TT counterparts provides an explanation for why they survive better when treated with dexamethasone than their non-TT counterparts (8). However, we had now shown in this study that among dexamethasone-treated tuberculous meningitis patients, *LTA4H* TT-independent cytokine increases are also associated with survival, raising the question of whether non-TT patients might also benefit from dexamethasone. By the time the cytokine analysis study was undertaken, adjunctive dexamethasone had become standard-of-care treatment so that all patients were given this drug (7). Therefore, to answer the question, we reanalyzed the survival data from the Tobin et al. (8) study (Table 1), using recently described Bayesian methods, which had compared survival of patients of the three *LTA4H* genotypes with and without adjunctive dexamethasone (8, 9).

**Table 1.** Patient cohort characteristics

	Thuong et al. (7) 2017	Tobin et al. (8) 2012
Total *n*	306	179
Age (y)		
Median (range)	39 (18–87)	34 (15–83)
BMRC tuberculous meningitis grade		
No. (% of total)		
1	109 (35.6)	43 (24.0)
2	141 (46.1)	84 (46.9)
3	56 (18.3)	52 (29.1)
Overall mortality		
No. (%)	57 (18.6)	38 (21.2)
LTA4H rs17525495 genotype		
No. (% of total)		
CC	148 (48.4)	84 (46.9)
CT	142 (46.4)	73 (40.8)
TT	16 (5.2)	22 (12.3)

**Fig. 4.**
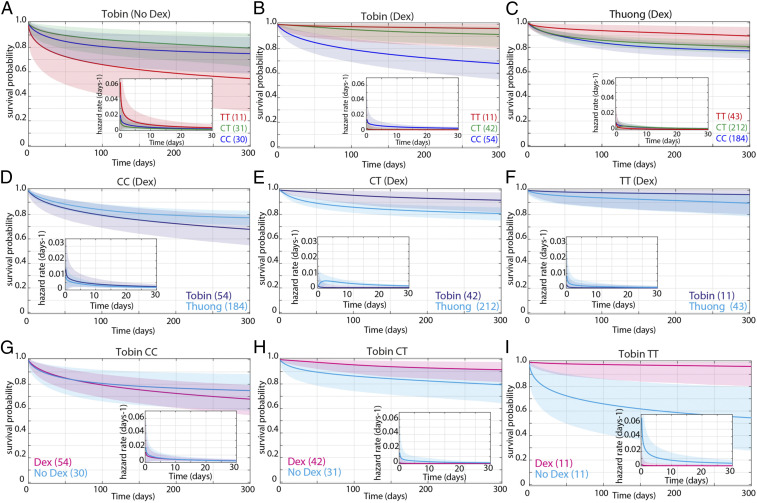
Effect of dexamethasone and *LTA4H* rs17525495 genotype on survival probability of patients from Tobin et al. (8) and Thuong et al. (7). Mean posterior survival probability curves; inset plots represent mean posterior hazard rates for the first 30 d. Shaded areas represent the 95% Bayesian confidence limits for posterior probability. (*A*) In Tobin et al. (8) (No Dex), TT survival was nonsignificantly reduced compared to non-TT (maximum probability 0.946). (*B*) In Tobin et al. (8) (Dex), TT survival was significantly greater than non-TT from day 40 onwards (maximum probability 0.976, survival gap 17%). Probability that TT hazard rate (*Inset*) is lower than non-TT is >0.95 from day 2 to day 252 (maximum probability 0.972, peak ratio 7.5 at day 97). CT survival was significantly greater than CC from day 3 onwards (maximum probability 0.999, survival gap 23%), and CT hazard rate significantly lower than CC from day 1 onwards (maximum probability 0.996, ratio peaks at 12 on day 3 and remains >3 throughout). (*C*) In Thuong et al. (7) patients (Dex), CC and CT survival comparisons do not differ significantly (maximum probability 0.91). TT survival was significantly greater than CC from day 42 onwards (maximum probability 0.987, survival gap 12%). Probability that TT hazard rate is lower than CC is >0.95 days 15 to 138 (maximum probability 0.991, ratio peaks at 3.4 on day 62 and remains >1 until day 250). TT survival was also significantly greater than CT from day 53 to day 254 (maximum probability 0.964, survival gap 9%). Probability that TT hazard rate is lower than CT is >0.95 from day 7 to day 73 (maximum probability 0.979, ratio peaks at 2.9 on day 22 and remains >1 to day 234). (*D*) In CC (+Dex) patients, survival was nonsignificantly greater in the Thuong et al. (7) cohort (maximum probability 0.939, survival gap 9%). (*E*) In CT (+Dex) patients, survival was significantly greater in the Tobin et al. (8) cohort from day 5 onwards (maximum probability 0.993, survival gap 11%). Tobin CT (+Dex) hazard rate was significantly lower than Thuong CT from day 2 to day 45 (maximum probability 0.997, peak ratio 9.8 on day 4). (*F*) In TT patients (+Dex), survival was nonsignificantly greater in the Tobin et al. (8) cohort (maximum probability 0.90, survival gap 6%). (*G*) Tobin CC patient survival did not differ significantly with and without Dex treatment (maximum probability 0.80). (*H*) Tobin CT patient survival was significantly greater with Dex from day 7 to day 88 (maximum probability 0.964, survival gap 11%). CT (+Dex) hazard rate was significantly lower than CT (No Dex) from day 3 to day 18 (maximum probability 0.967, peak ratio 9 on day 2 and remains >1 throughout). (*I*) Tobin TT patient survival was significantly greater with Dex from day 1 onwards (maximum probability 0.997, survival gap 41%). TT (+Dex) hazard rate was significantly lower from day 1 onwards (maximum probability 0.996, peak ratio 35 on day 2). See *SI Appendix*, Supplementary Box S2 for explanation of definitions and abbreviations used.

The survival of dexamethasone-treated CT heterozygotes was different between the two studies, appearing more similar to that of the TT patients in the Tobin et al. study (8) but more similar to that of the CC patients in the Thuong et al. study (7). Therefore, we reanalyzed both studies using Bayesian methods, separating the non-TT patients into the individual CC and CT genotypes. In the Tobin et al. study (8), in the absence of dexamethasone treatment, TT patients had worse survival than both CC and CT patients, with the difference being just short of being significant (maximum posterior probability 0.946) (Fig. 4*A*; also see *SI Appendix*, Supplementary Box S2 for explanation of definitions and abbreviations used in the Fig. 4 legend). There was no significant difference between CC and CT patients (Fig. 4*A*). Among dexamethasone-treated patients, TT survival was significantly higher than CC survival (Fig. 4*B*). CT survival was in between the two, significantly higher than CC and nonsignificantly lower than TT (Fig. 4*B*). In the Thuong et al. study (7), CT survival was significantly worse than TT and not significantly different from CC (Fig. 4*C*). We confirmed this shift in CT survival between the two studies by a direct comparison of the Tobin et al. and Thuong et al. studies (7, 8). CT survival was significantly worse in the Thuong et al. study (7), whereas CC and TT survival were not significantly different in the two studies (Fig. 4 *D*–*F*).

Finally, we asked whether and how dexamethasone influenced the survival of each genotype in the Tobin et al. study (8). Directly comparing survival of each of the three genotypes with and without dexamethasone, we found that TT patients derived the greatest benefit from dexamethasone, CT patients had a smaller but still significant benefit, and CC patients were neither helped nor harmed by dexamethasone (Fig. 4 *G*–*I*).

**Fig. 5.**
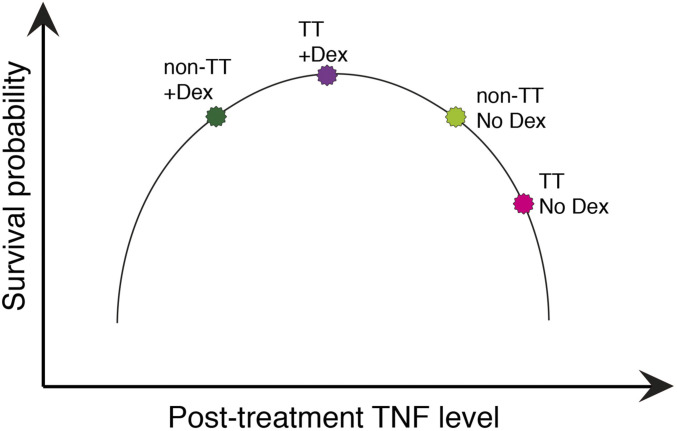
Proposed model of dexamethasone-mediated effects on survival probability interacting with *LTA4H* rs17525495 genotypes.

In sum, because the CC and TT patients survived similarly in response to dexamethasone in the Thuong et al. and Tobin et al. studies, we can use the survival with and without dexamethasone in the Tobin et al. study (8) together with the pretreatment cytokine levels in the Thuong et al. study (7) to draw the following two conclusions: 1) TT patients benefit very substantially from dexamethasone, consistent with their higher pretreatment cytokine levels; and 2) among dexamethasone-treated patients, CC survivors have higher pretreatment cytokine levels than nonsurvivors. These two findings can be reconciled by a model (Fig. 5) where dexamethasone reduces the higher pretreatment cytokine levels in TT patients to a level optimal for survival, whereas the lower cytokine pretreatment levels in many of the CC patients are lowered further by this treatment to suboptimal levels, so that there is no apparent benefit of the drug to the overall cohort.

## Discussion

Dysregulated intracerebral inflammation has long been thought to be responsible for the high mortality and morbidity of tuberculous meningitis. Multiple cytokines can be major effectors of dysregulated immune responses, yet pretreatment cytokine data from tuberculous meningitis patients are limited (18, 19). Therefore, the Thuong et al. study (7) where CSF cytokines were collected in 306 HIV-uninfected patients, all of whom were treated with dexamethasone, together with comprehensive clinical information and survival analyses, provided an unprecedented opportunity. This cohort allowed for an analysis of CSF cytokine concentrations in tuberculous meningitis with respect to disease severity on presentation and outcome following dexamethasone treatment. Moreover, this study confirmed the survival benefit of the *LTA4H* TT genotype, providing the opportunity to ask if this hyperinflammatory genotype was associated with increased cytokines. Comparison of CSF cytokines applying frequentist statistical methods (linear trends tests) to median cytokine values showed that increased pretreatment levels of most (8 of the 10 tested) were significantly associated with survival with dexamethasone, indicating that these higher levels are pathogenic. All cytokines were increased with increased disease grade, but only one of these, IFN-γ, was significantly increased in those with grade 3 disease. The *LTA4H* TT genotype was also associated with global increases in cytokine concentrations across the board in comparison to the non-TT genotype patients, but the differences were significant in only 3 of the 10 cytokines. Given that most cytokines are induced by interrelated and often shared signal transduction networks, notably the NF-κB family of transcription factors (20), these patchy statistically significant differences were more likely to represent a type 2 statistical error than biologically relevant patterns. Therefore, we turned to Bayesian methods to reanalyze these data and looked for associations between pretreatment cytokine levels with disease severity, survival and *LTA4H* genotype, not only singly, but also in combination.

Bayesian analysis shows that all cytokines tested, representing multiple functional classes—innate proinflammatory, Th1- and Th2-associated, and immunomodulatory—are associated with survival in this dexamethasone-treated cohort. This global induction of cytokines is consistent with the induced inflammatory trigger mediating effects upstream of a common signaling axis for all of them without significant additional downstream regulation. The finding that the *LTA4H* TT genotype further increases all 10 cytokines is consistent with prior work showing that LTB_4_ binding to its receptors activates the NF-κB pathway (21). This enhancement in NF-κB activity may not only boost the transcription of inflammatory cytokines, but may also induce immunomodulatory cytokines. For example, the *IL-10* locus contains an NF-κB binding motif that enhances *IL-10* transcription in macrophages, cells with a critical, early involvement in tuberculosis pathogenesis (8, 12, 22). Importantly, TNF, which has been implicated in the pathogenesis of tuberculosis, including tuberculous meningitis (13, 14, 23), appears to be dysfunctionally increased both in an *LTA4H*-independent and -dependent manner. Finally, this work highlights the role of the previously described regulatory circuits that dampen *LTA4H* TT-mediated inflammation in the context of one of the most lethal infectious diseases of humans (11, 24).

### Association of Increased CSF Cytokines and Survival even Independent of *LTA4H* Genotype.

Our initial goal in performing these analyses was to ask whether the *LTA4H* TT genotype is associated with global increases in pretreatment CSF cytokines, as would be predicted by its activation of the NF-κB pathway (21). *LTA4H* TT individuals with tuberculous meningitis have a striking survival benefit from dexamethasone, which causes a global reduction in cytokines, and we find that the TT genotype is indeed associated with higher pretreatment CSF cytokines across the board. However, even in Europe and Africa where the *LTA4H* T allele is much rarer (10% frequency) than in Asia (up to 33%; from https://tinyurl.com/y4c232e3) (8, 9, 25), multiple small studies have a modest survival benefit from corticosteroids comparable to that seen in Asia (4, 5, 26, 27). In fact, the earliest studies suggesting corticosteroid benefit, which gave the impetus for the larger randomized controlled trial in Vietnam (6), were done in patient populations that were mostly of European descent, in which the *LTA4H *TT homozygote genotype frequency would have been rare (1 to 4% of population) (25–27). Perhaps our most important finding is that even among *LTA4H* non-TT individuals, higher CSF cytokines are associated with higher survival in response to dexamethasone. However, when we analyze a prior cohort that enables comparison of survival with and without dexamethasone (but lacks CSF cytokine analysis), we find that while TT patients gain a major survival advantage from dexamethasone, non-TT patients’ survival is neither helped nor harmed by it.

These two findings can be reconciled in two ways. The first is predicated on the idea that a major mechanism of dexamethasone’s survival benefit is through its cytokine-reducing effect (28, 29). Non-TT pretreatment CSF cytokines are lower on average than TT, and the currently used dexamethasone dosages may decrease their cytokine levels to suboptimal levels (Fig. 5). In optimal concentrations, many of these cytokines play a role in the host responses that eliminate bacteria, and this function may be lost if they are lowered below a threshold. The model that dexamethasone optimizes TT cytokine levels while reducing non-TT levels too much can be tested when the results of an ongoing randomized clinical trial of dexamethasone for non-TT patients become available (trial registration: NCT03100786) (30). Non-TT survivors in the control arm would not be expected to have higher cytokine levels than nonsurvivors. Our findings could pave the way for stratification of patients for dexamethasone therapy both by *LTA4H* genotype and by CSF cytokine levels, potentially indicating higher and lower doses of the drug or no drug at all. Because corticosteroids exhibit a dose-dependent degree of immunosuppression of cytokines, including TNF (31), it is possible that the lower non-TT grades may benefit from lower doses of these drugs.

Alternatively, it is possible that the much greater benefit of dexamethasone on TT survival derives from its countering other inflammatory pathways activated by LTB_4_, including neutrophil chemotaxis and degranulation, and production of reactive oxygen and nitrogen species (10), which would also be inhibited by corticosteroids (28, 29).

### Regulatory Networks May Keep *LTA4H *TT Cytokine Increases in Check.

The finding that disease severity increases cytokine levels in non-TT but not in TT patients suggests *LTA4H* TT-specific compensatory mechanisms that appear to dampen disease grade mediated cytokine increases. LTB_4_ mediates its activity through two receptors, BLT1 and BLT2 (32). BLT1, the high-affinity receptor, is associated with proinflammatory responses, and is itself down-regulated by increased inflammatory determinants, including TNF (11, 24). One could imagine a scenario where the interplay between disease severity and TT-driven cytokine increases are sufficiently high so as to down-regulate BLT1, which would halt LTB_4_–BLT1-mediated cytokine increases. Moreover, BLT1 down-regulation would promote LTB_4_ interactions with its BLT2 receptor which, having an ∼50-fold lower affinity (32), would ordinarily not be in play. LTB_4_–BLT2 interactions can promote both pro- and antiinflammatory responses in different scenarios (11, 24, 32, 33). Germane to this study, LTB_4_–BLT2 interactions are reported to down-regulate macrophage activation as well as all four cytokines tested (TNF, IL-1β and IL-6, and IFN-γ) in a mouse inflammatory colitis model (33). Even if the interaction results in proinflammatory responses, the substantial reduction in binding affinity would be expected to result in reduced downstream effects. We hope to be able to evaluate these potential compensatory mechanisms further in both zebrafish and human studies.

### *LTA4H *TT-Dependent and *LTA4H*-Independent TNF Dysregulation.

Our finding that dexamethasone is associated with increased survival in both *LTA4H *TT and non-TT patients is tantalizing from a therapeutic standpoint. In the zebrafish, high LTA4H increases disease severity through increasing TNF, which in excess causes increased disease pathogenesis through a newly identified programmed macrophage necrosis (13, 14). We were particularly interested in this question because in the zebrafish, several pathway-specific drugs that inhibit macrophage necrosis without being broadly antiinflammatory have been identified, all of which have a decades-long history of use in humans for other conditions (13, 14). Therefore, unlike glucocorticoids, these drugs would be beneficial to those with excessive TNF while being neutral to the other patients, as they target the downstream effects of excess TNF, without broadly reducing overall cytokines to levels which are detrimental to survival.

### Conclusions and Implications for Future Studies.

The use of Bayesian methods has enabled important insights into the induction and regulation in tuberculous meningitis and the possible detrimental effects of their dysregulation. On-going randomized control trials that will enroll >1,200 participants are examining the role of adjunctive dexamethasone adults with tuberculous meningitis, stratifying participants according to HIV infection and *LTA4H* genotype (30, 34). Pre- and posttreatment CSF cytokine analysis will be performed in all participants (30, 34). These trials will allow for validation of the analyses presented here as well as test the models and hypotheses that have arisen from them. Finally, some (though not all) studies done over decades across the globe have found adjunctive corticosteroid treatment to have a modest early benefit in the contagious and most common form of tuberculosis, that involving the lung, both in reducing inflammation and bacterial burdens (35–38). Spatial studies of human tuberculous granulomas find that necrotic tuberculous granulomas are enriched for LTA4H and TNF as compared to nonnecrotic granulomas from the same lung, making it plausible that corticosteroids exert their effects through these determinants (39). The analytical methods developed here could be readily tailored to examine the role of corticosteroids as drugs that may improve outcome in pulmonary TB as well.

## Materials and Methods

The anonymized tuberculous meningitis patient cohort CSF cytokine-level data used here has been previously described (7, 9) (Table 1 and Dataset S1). The tuberculous meningitis cohort used for survival analysis has also been previously described (6, 8) (Table 1 and Dataset S1). Patients were admitted to one of two tertiary care referral hospitals in Ho Chi Minh City, Vietnam: Pham Ngoc Thach Hospital for Tuberculosis and Lung Disease (Hospital 1) or the Hospital for Tropical Diseases (Hospital 2). Patients were grouped on study entry according to the modified BMRC tuberculous meningitis grade: Patients with a Glasgow Coma Scale (GCS) score of 15, with no focal neurologic signs, were designated grade 1; patients with GCS score of either 11 to 14, or 15 with focal neurologic signs, were designated as grade 2; and patients with GCS scores of 10 or less were designated as grade 3 (6). The Tobin et al. (8) study had a higher proportion of patients with more severe disease (Table 1 and Dataset S1). Patients with cytokine measurements were classified as having definite, probable, or possible tuberculous meningitis in accordance with published diagnostic criteria (40). Patients in the Tobin et al.(8) study all had definite tuberculous meningitis. *LTA4H* rs17525495 genotypes were determined by Taqman assay (7, 8). There was no significant difference in the frequency of the three *LTA4H* genotypes, CC, CT, and TT between the Thuong et al. (7) and Tobin et al. (8) patients when the comparisons were restricted to the individual grades. When all grades were combined, Thuong et al. (7) had more CT patients (*P* 0.96), Tobin et al. (8) had more CC patients with the difference being not quite significant, and there was no difference between the TT patients in the two studies.

The Tobin et al. (8) study used for survival analysis comprised patients from a trial where patients were randomized to get either dexamethasone for the first 6 to 8 wk or placebo (6, 8). Patients with moderate to severe disease (grades 2 and 3) were given intravenous treatment for 4 wk (0.4 mg/kg/d week 1, 0.3 mg/kg/d week 2, 0.2 mg/kg/d week 3, and 0.1 mg/kg/d week 4), followed by oral administration for 4 wk, starting at 4 mg/d and decreasing by 1 mg each week. Patients with mild disease (grade 1) received 2 wk of intravenous treatment (0.3 mg/kg/d week 1, 0.2 mg/kg/d week 2), followed by oral therapy for 4 wk, starting at 0.1 mg/kg/d in week 3, then a total of 3 mg/d in week 4, decreasing by 1 mg/week. All patients in the cohort used for cytokine analysis were treated with adjunctive dexamethasone as above. CSF specimens had been collected on enrollment and cytokines had been measured in 306 of 404 patients (7) (Dataset S1). CSF for cytokine measurements was available in a smaller proportion of patients admitted to Hospital 1 than Hospital 2 (162 of 190 [55.9%] vs. 144 of 149 [96.6%]) (*SI Appendix*, Table S1).

Further analysis showed that Hospital 1 TT patients were significantly less likely to have had cytokine measurements than non-TT patients (*SI Appendix*, Table S2). There was no such bias in relation to survival status or disease grade severity (*SI Appendix*, Table S2). To determine if the Hospital 1 collection bias in the TT vs. non-TT patients changed the analysis, all data were analyzed for the whole cohort and Hospital 2 alone (Dataset S2). Of the 350 comparisons, 349 yielded similar patterns in the two datasets, with some loss of significance when considering Hospital 2 alone due to reduced patient numbers. The only comparison where there was a change in the direction was IL-5, grade 1 versus grade 2, which was significantly greater in grade 2 than in grade 1 in Hospital 2 alone, and nonsignificantly greater in grade 1 than grade 2 in the overall cohort. The analyses for the overall cohort are presented in Figs. 1–3.

As described in Thuong et al. (7), 10 cytokines (TNF, IL-1β, IL-6, IL-12, IFN-γ, IL-2, IL-4, IL-5, IL-13, and IL-10) were measured in the stored CSF samples by Luminex multiplex bead array analysis. The cytokine levels reported in the previous study were reanalyzed using Bayesian methods comparing restricted geometric mean values from groups of patients separated by HIV status, survival status (“survivors” are patients who were either known to have survived or, if lost to follow-up, were censored at the time of last recorded outcome), BMRC tuberculous meningitis grade, and *LTA4H* rs17525495 genotype (*SI Appendix*, Appendix S2). A number of CSF samples yielded cytokine concentrations that were outside the linear ranges of the assays and had been assigned specified high or low fixed values. To handle apparent limits to the measurable value, resulting in many observations of the maximum and minimum seen value, these maximal and minimal values were spread in a process called dithering (*SI Appendix*, Appendix S2). The geometric means of both the undithered and dithered data are in *SI Appendix*, Table S2. The geometric means indicated in Figs. 1–3 are based on the undithered data. Significance analyses for all comparisons were performed on both dithered and undithered data (Dataset S2) and the results from the analyses of the dithered data are presented in Figs. 1–3. In 869 of the 890 comparisons, the significance for the majority of comparisons did not change between analysis of undithered and dithered data. Of the 21 comparisons that were changed by dithering, significance was lost upon dithering in 15 and gained upon dithering in 6. The instances where significance changed upon dithering are color-coded blue and marked with a minus sign in the less-significant cell in Dataset S2.

The Bayesian methods used for the survival analysis in Fig. 4 are detailed in Whitworth et al. (9).

## Supplementary Material

Supplementary File

Supplementary File

Supplementary File

Supplementary File

## Data Availability

All study data are included in the article and supporting information.

## Change History

September 29, 2021: Figure 3 has been updated; please see accompanying Correction for details.

## References

[1] A. M. Stadelman., Treatment outcomes in adult tuberculous meningitis: A systematic review and meta-analysis. Open Forum Infect. Dis. 7, ofaa257 (2020). 32818138 10.1093/ofid/ofaa257 PMC7423296

[2] A. van Laarhoven., Clinical parameters, routine inflammatory markers, and LTA4H genotype as predictors of mortality among 608 patients with tuberculous meningitis in Indonesia. J. Infect. Dis. 215, 1029–1039 (2017). 28419315 10.1093/infdis/jix051

[3] R. J. Wilkinson.; Tuberculous Meningitis International Research Consortium, Tuberculous meningitis. Nat. Rev. Neurol. 13, 581–598 (2017). 28884751 10.1038/nrneurol.2017.120

[4] K. Prasad, M. B. Singh, H. Ryan, Corticosteroids for managing tuberculous meningitis. Cochrane Database Syst. Rev. 4, CD002244 (2016). 27121755 10.1002/14651858.CD002244.pub4 PMC4916936

[5] J. F. Schoeman, J. W. Elshof, J. A. Laubscher, A. Janse van Rensburg, P. R. Donald, The effect of adjuvant steroid treatment on serial cerebrospinal fluid changes in tuberculous meningitis. Ann. Trop. Paediatr. 21, 299–305 (2001). 11732147 10.1080/07430170120093481

[6] G. E. Thwaites., Dexamethasone for the treatment of tuberculous meningitis in adolescents and adults. N. Engl. J. Med. 351, 1741–1751 (2004). 15496623 10.1056/NEJMoa040573

[7] N. T. T. Thuong., Leukotriene A4 hydrolase genotype and HIV infection influence intracerebral inflammation and survival from tuberculous meningitis. J. Infect. Dis. 215, 1020–1028 (2017). 28419368 10.1093/infdis/jix050 PMC5426373

[8] D. M. Tobin., Host genotype-specific therapies can optimize the inflammatory response to mycobacterial infections. Cell 148, 434–446 (2012). 22304914 10.1016/j.cell.2011.12.023 PMC3433720

[9] L. Whitworth., A Bayesian analysis of the association between *Leukotriene A4 Hydrolase* genotype and survival probability of tuberculous meningitis patients treated with adjunctive dexamethasone. elife 10, e61722 (2021). 33416499 10.7554/eLife.61722 PMC7793626

[10] S. L. Brandt, C. H. Serezani, Too much of a good thing: How modulating LTB_4_ actions restore host defense in homeostasis or disease. Semin. Immunol. 33, 37–43 (2017). 29042027 10.1016/j.smim.2017.08.006 PMC5679129

[11] H. Qiu., Differential induction of BLT receptor expression on human endothelial cells by lipopolysaccharide, cytokines, and leukotriene B4. Proc. Natl. Acad. Sci. U.S.A. 103, 6913–6918 (2006). 16624877 10.1073/pnas.0602208103 PMC1440767

[12] D. M. Tobin., The lta4h locus modulates susceptibility to mycobacterial infection in zebrafish and humans. Cell 140, 717–730 (2010). 20211140 10.1016/j.cell.2010.02.013 PMC2907082

[13] F. J. Roca, L. Ramakrishnan, TNF dually mediates resistance and susceptibility to mycobacteria via mitochondrial reactive oxygen species. Cell 153, 521–534 (2013). 23582643 10.1016/j.cell.2013.03.022 PMC3790588

[14] F. J. Roca, L. J. Whitworth, S. Redmond, A. A. Jones, L. Ramakrishnan, TNF induces pathogenic programmed macrophage necrosis in tuberculosis through a mitochondrial-lysosomal-endoplasmic reticulum circuit. Cell 178, 1344–1361.e11 (2019). 31474371 10.1016/j.cell.2019.08.004 PMC6736209

[15] D. K. Dastur, V. S. Lalitha, P. M. Udani, U. Parekh, The brain and meninges in tuberculous meningitis-gross pathology in 100 cases and pathogenesis. Neurol. India 18, 86–100 (1970). 5459296

[16] D. K. Dastur, D. K. Manghani, P. M. Udani, Pathology and pathogenetic mechanisms in neurotuberculosis. Radiol. Clin. North Am. 33, 733–752 (1995). 7610242

[17] F. Brancusi, J. Farrar, D. Heemskerk, Tuberculous meningitis in adults: A review of a decade of developments focusing on prognostic factors for outcome. Future Microbiol. 7, 1101–1116 (2012). 22953709 10.2217/fmb.12.86

[18] P. R. Donald., Concentrations of interferon gamma, tumor necrosis factor alpha, and interleukin-1 beta in the cerebrospinal fluid of children treated for tuberculous meningitis. Clin. Infect. Dis. 21, 924–929 (1995). 8645841 10.1093/clinids/21.4.924

[19] C. P. Simmons., The clinical benefit of adjunctive dexamethasone in tuberculous meningitis is not associated with measurable attenuation of peripheral or local immune responses. J. Immunol. 175, 579–590 (2005). 15972695 10.4049/jimmunol.175.1.579

[20] P. P. Tak, G. S. Firestein, NF-kappaB: A key role in inflammatory diseases. J. Clin. Invest. 107, 7–11 (2001). 11134171 10.1172/JCI11830 PMC198552

[21] E. Sánchez-Galán., Leukotriene B4 enhances the activity of nuclear factor-kappaB pathway through BLT1 and BLT2 receptors in atherosclerosis. Cardiovasc. Res. 81, 216–225 (2009). 18852255 10.1093/cvr/cvn277

[22] M. Saraiva., Identification of a macrophage-specific chromatin signature in the IL-10 locus. J. Immunol. 175, 1041–1046 (2005). 16002704 10.4049/jimmunol.175.2.1041

[23] L. Tsenova, A. Bergtold, V. H. Freedman, R. A. Young, G. Kaplan, Tumor necrosis factor alpha is a determinant of pathogenesis and disease progression in mycobacterial infection in the central nervous system. Proc. Natl. Acad. Sci. U.S.A. 96, 5657–5662 (1999). 10318940 10.1073/pnas.96.10.5657 PMC21916

[24] A. Pettersson., Pro- and anti-inflammatory substances modulate expression of the leukotriene B4 receptor, BLT1, in human monocytes. J. Leukoc. Biol. 77, 1018–1025 (2005). 15728714 10.1189/jlb.1204740

[25] A. Auton.; 1000 Genomes Project Consortium, A global reference for human genetic variation. Nature 526, 68–74 (2015). 26432245 10.1038/nature15393 PMC4750478

[26] M. Ashby, H. Grant, Tuberculous meningitis treated with cortisone. Lancet 268, 65–66 (1955). 13222850 10.1016/s0140-6736(55)90003-9

[27] S. J. Shane, C. Riley, Tuberculous meningitis: Combined therapy with cortisone and antimicrobial agents. N. Engl. J. Med. 249, 829–834 (1953). 13111361 10.1056/NEJM195311192492101

[28] D. Annane, J. M. Cavaillon, Corticosteroids in sepsis: From bench to bedside? Shock 20, 197–207 (2003). 12923489 10.1097/01.shk.0000079423.72656.2f

[29] M. Zen., The kaleidoscope of glucorticoid effects on immune system. Autoimmun. Rev. 10, 305–310 (2011). 21224015 10.1016/j.autrev.2010.11.009

[30] J. Donovan., Adjunctive dexamethasone for the treatment of HIV-uninfected adults with tuberculous meningitis stratified by Leukotriene A4 hydrolase genotype (LAST ACT): Study protocol for a randomised double blind placebo controlled non-inferiority trial. Wellcome Open Res. 3, 32 (2018). 30363837 10.12688/wellcomeopenres.14007.1 PMC6182672

[31] M. D. de Kruif., Prednisolone dose-dependently influences inflammation and coagulation during human endotoxemia. J. Immunol. 178, 1845–1851 (2007). 17237435 10.4049/jimmunol.178.3.1845

[32] A. M. Tager, A. D. Luster, BLT1 and BLT2: The leukotriene B(4) receptors. Prostaglandins Leukot. Essent. Fatty Acids 69, 123–134 (2003). 12895595 10.1016/s0952-3278(03)00073-5

[33] Y. Iizuka., Protective role of the leukotriene B4 receptor BLT2 in murine inflammatory colitis. FASEB J. 24, 4678–4690 (2010). 20667973 10.1096/fj.10-165050

[34] J. Donovan., Adjunctive dexamethasone for the treatment of HIV-infected adults with tuberculous meningitis (ACT HIV): Study protocol for a randomised controlled trial. Wellcome Open Res. 3, 31 (2018). 30320225 10.12688/wellcomeopenres.14006.1 PMC6143919

[35] S. Bilaçeroğlu, K. Perim, M. Büyükşirin, E. Celikten, Prednisolone: A beneficial and safe adjunct to antituberculosis treatment? A randomized controlled trial. Int. J. Tuberc. Lung Dis. 3, 47–54 (1999). 10094169

[36] H. Mayanja-Kizza.; Uganda-Case Western Research Collaboration, Immunoadjuvant prednisolone therapy for HIV-associated tuberculosis: A phase 2 clinical trial in Uganda. J. Infect. Dis. 191, 856–865 (2005). 15717259 10.1086/427995 PMC4515766

[37] P. Muthuswamy, T. C. Hu, B. Carasso, M. Antonio, N. Dandamudi, Prednisone as adjunctive therapy in the management of pulmonary tuberculosis. Report of 12 cases and review of the literature. Chest 107, 1621–1630 (1995). 7781357 10.1378/chest.107.6.1621

[38] R. A. Smego, N. Ahmed, A systematic review of the adjunctive use of systemic corticosteroids for pulmonary tuberculosis. Int. J. Tuberc. Lung Dis. 7, 208–213 (2003). 12661833

[39] M. J. Marakalala., Inflammatory signaling in human tuberculosis granulomas is spatially organized. Nat. Med. 22, 531–538 (2016). 27043495 10.1038/nm.4073 PMC4860068

[40] S. Marais., Tuberculous meningitis: A uniform case definition for use in clinical research. Lancet Infect. Dis. 10, 803–812 (2010). 20822958 10.1016/S1473-3099(10)70138-9

