# Supplementary material for: Elevated cerebrospinal fluid cytokine levels in tuberculous meningitis predict survival in response to dexamethasone
Source: Proc Natl Acad Sci U S A. 2021 Mar 3;118(10):e2024852118. doi: 10.1073/pnas.2024852118 (PMC7958233; doi:10.1073/pnas.2024852118)
Supplement: Supplementary File [file pnas.2024852118.sapp.pdf]

Supplementary Box 1

Definitions and usages

Definitions

- *Posterior probability* – the probability after seeing the data
- *Survivors* (as opposed to nonsurvivors) – those who were not known to have died during the period that they were observed, i.e. those who either were censored or died.
- *Restricted geometric mean* – the geometric mean of a distribution, restricted to an interval excluding only extreme tail events; see Appendix 2 for reasons for using this concept
- *Dithering* – A process that spreads out data points concentrated on the maximum and minimum seen values, as these likely come from a limit in the measurement procedure

Abbreviated and example for Figures 1-3 and associated text

"A was significantly greater than B" - the posterior probability given the dithered data that the restricted geometric mean of the variable A in the relevant population was greater than that of the variable B was at least 0.95 (and similarly for "*significant increase*", etc.)

"A was not significantly different from B" - the posterior probability given the dithered data that the restricted geometric mean of the variable A in the relevant population was greater than that of the variable B was between 0.05 and 0.95 (and similarly for "*no significant difference*", etc.)

"*A was greater than B*" - the (restricted) geometric mean of the undithered data in A was greater that of B (note that the presence or absence of "restricted" makes no difference in this definition as all the observed data is within the range of restriction)

"*The probability that A was greater than B was p*" - the posterior probability given the dithered data that the restricted geometric mean of the variable A in the relevant population was greater than that of the variable B was p

Supplementary Box 2

Definitions - Figure 4 survival analysis

- *Posterior probability* - the probability after seeing the data
- *Mean posterior survival probability at time T* - the expectation after seeing the data of the fraction of patients that will still be alive at time T
- *Hazard rate* - the fraction of those still surviving that will die per unit time. A high hazard rate at a particular time indicates that patients are at high risk of dying at that time
- *Mean posterior hazard rate at time T* **-** the expectation after seeing the data of the hazard rate at time T

Abbreviated and example usages

Onwards - for the rest of the 270-day observation period

Throughout - for the entire 270-day observation period

"A was significantly greater than B at time T" - the posterior probability that A was greater than B, at time T, was at least 0.95

"A was not significantly different from B" - the posterior probability that A was greater than B was between 0.05 and 0.95 throughout

"Group A survival was 30% greater than group B" - the mean posterior survival probability at 270 days, p_A, was 30% absolute greater than the corresponding probability p_B for group B. ("*absolute*" here meaning that $p_A = p_B + 0.3$, and not that $p_A = p_B \times 1.3$)

"Probability that group A survival was better than group B at time T was 0.97" - the posterior probability that group A survival probability at time T was greater than group B survival probability at time T was 0.97 (Note that this is not a reference to the mean posterior survival probability.)

"The hazard rate ratio for group A over group B peaked at X at time T and remained greater than Y throughout" or *"Group A had an X-fold higher relative risk of death at time T which remained greater than Y throughout"* - the mean posterior hazard rate for group A, divided by that for group B, peaked at a value of X at time T and remained greater than Y at all times up to 270 days.

"The probability that hazard rate for group A was greater than that of group B was 0.97 at time T" - the posterior probability was 0.97 that the hazard rate for group A at time T was greater than that for group B at time T (Note that this is not a reference to the mean posterior hazard rate.)

"*The hazard rate for group A was greater than that for group B at time T*" - the mean posterior hazard rate for group A was greater than that for group B at time T.

"*survival gap"* - the difference in mean posterior survival probability between the two groups being considered at 270 days

Supplementary Table 1. Thuong 2017 cohort separated by hospital

	All of Thuong 2017 cohort (n= 439)	
	**Hospital 1**	**Hospital 2**
**Total n**	290	149
**Age in years** *median (range)*	44 (18-93)	35 (18-86)
**BMRC TBM grade** *no. (% of total)*		
**1**	118 (40.7)	45 (30.2)
**2**	133 (45.9)	73 (49.0)
**3**	39 (13.4)	31 (20.8)
**Overall mortality**	63 (23.5)	20 (13.4)
**Patients with cytokine data**	162 (55.9)	144 (96.6)

Supplementary Table 2. Probabilities of selection bias in Hospital 1

Comparison Groups	Probability that first group more likely to have cytokine data than second group
**CC than non-CC**	**0.999991**
**CT than non-CT**	0.21
**TT than non-TT**	**3.2×10^{-9}**
**Survivors than Nonsurvivors**	0.31
**BMRC Grade 1 than Grades 2 and 3**	0.32
**BMRC Grade 2 than Grades 1 and 3**	0.29
**BMRC Grade 3 than Grades 1 and 2**	0.92

Supplementary Figure 1.

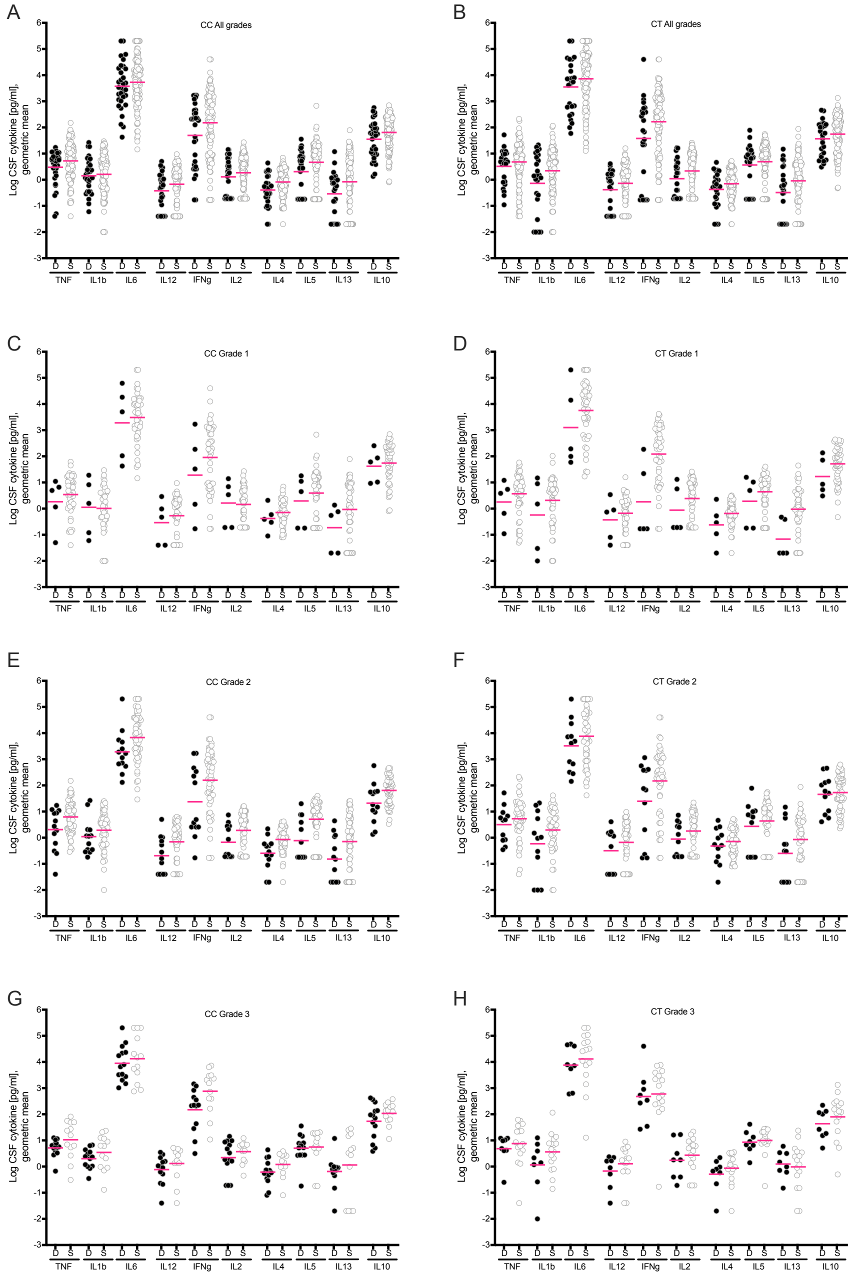

Supplementary Figure 1. Cytokine levels in HIV-negative CC and CT patients, survivors and non-survivors. (A, B) Cytokine levels in patients who died (D) versus those who survived (S), in CC patients (deaths n=32, survivors n=116) and CT patients (deaths n=25, survivors n=117). (C, D) Cytokine levels in Grade 1 CC (deaths n=5, survivors n=46) and CT (deaths n=5, survivors n=49) patients. (E, F) Cytokine levels in Grade 2 CC (deaths n=13, survivors n= 57) and CT (deaths n=12, survivors n=51) patients. (G, H) Cytokine levels in Grade 3 CC (deaths n=14, survivors n=13) and CT (deaths n=8, survivors n=16) patients. Magenta lines indicate geometric means of non-dithered data. Statistical comparisons of the geometric means were not calculated for these subsets.

Appendix 1
An intuitive description of the distribution comparison problem

Contents

1 Introduction 1

2 An intuitive way of thinking about the distribution comparison problem 1

3 Some warnings 2

1 Introduction

This appendix aims to give an intuitive introduction, for those who haven't encountered Bayesian methods before, to how to think about the distribution comparison problem: given two sets of numbers, how to work out the probability that the numbers in one set are in some sense bigger than those in the other set, when both are distributed according to members of the same, unknown, family of probability distributions.

2 An intuitive way of thinking about the distribution comparison problem

We will be considering various possible families of probability distributions that might describe our data, and within each family, various specific distributions. The reader may perhaps be thinking "we should fit probability density functions to histograms of the data", but we would like to *dissuade* the reader from doing this, and instead adopt the viewpoint of a detective who is trying to work out in retrospect how nature did something.

We think of the data generation process as follows: Assume nature has a kit-bag F of various families of probability distributions it can use. To generate the dataset for a particular cytokine for a subset S of the patients, nature first picks a distribution family f out of its kit-bag. That distribution family has some parameters; e.g. the Gaussian has μ and σ (mean and standard deviation) in the most commonly used parameterisation, while the log-skew-Student has four parameters as we shall see.

So nature next picks a set of parameters θ_f for f, getting a specific distribution. Having done that, some "simple" calculation could (if we had the information on f and θ_f) tell us for example the median M_S of that distribution.

Finally, nature draws random values (one for each patient in the subset) from that distribution to give the data A for that cytokine for that subset S of the patients.

Nature then does the same thing for another subset T of the patients, leaving the investigator with two sets of real numbers A and B representing cytokine levels in the two subsets of patients S and T respectively. Nature also knows (but the investigator doesn't) the values M_S and M_T.

The investigator is then cast in the role of detective who wants to know whether M_S or M_T is greater, and how sure he can be of this answer. Bayesian methods then tell him that his first step is to decide how likely it was — before seeing the data ! — that nature picked each of the distribution families in F, and his second is to decide how likely it was for each f in F that nature picked each particular possible set of parameters θ_f.

Having done that, some calculations in probability theory enable him to work out what is the probability, given the data, that M_S is bigger than M_T. We never have to use traditional curve-fitting, but of course do end up with a belief in the distribution of the observations. Note that because we are dealing here exclusively with continuous distributions, the probability that $M_S = M_T$ exactly is zero — there will always be some small difference between the two, and we may or may not be able to be sure which way round that difference is.

Note also that in what follows we will be assuming that nature has used the *same* family f for making both A and B, it is just the parameter values that are different between the two subsets.

3 Some warnings

In general Bayesian inference starts from a prior probability distribution on some unknown variables, collects some data, and calculates the posterior distribution on those variables, i.e. the probability distribution that tells us what we then know about those variables. Typically, as here, that is a distribution on a vector of variables containing more than one variable.

In this situation, there are a number of pitfalls for the unwary; we mention the following:

1. It may be tempting to suppose that we should just report the value of the variables at the point where the posterior probability density is greatest; this point is known as the *maximum a posteriori* (MAP) value, and is the mode of the posterior distribution.

   However, unless we are in the very special situation that any error is equally bad as any other[1], this is usually a really bad idea, despite the fact that it has been taught for decades in engineering departments. There are several reasons for this:

   (a) For a single variable x, and a function g, the mode of the distribution of the function of x is usually *not* at the function of the mode of the distribution of x. A good example of this with $g(x) = \log(x)$ is if
   $$P(x) = 2(1-x)[x \in (0,1)]$$
   and $y = \log(x)$, in which case
   $$P(y) = 2e^y(1-e^y)[y < 0].$$
   Then the mode of x is at 0 but the mode of y is at $-0.693 \approx -\log(2) \neq \log(0) = -\infty$.

   (b) If the mode of the distribution of (μ, m, s) is at $(\hat{\mu}, \hat{m}, \hat{s})$, then the mode of the resulting marginal distribution of μ is usually *not* at $\hat{\mu}$. A 2-d rather than 3-d example of this is shown in Figure 1, where the mode of x is at about 2.09, well to the left of the mode of (x, y) (whose x-coordinate is at 3.55).

[1] In most situations different errors carry differing importance — e.g. concluding that x is 10 times its true value is worse than concluding that it is twice its true value. Similarly concluding that a distribution, that is in truth uniform on an interval, is Gaussian would usually be a less bad error than concluding that it is Cauchy.

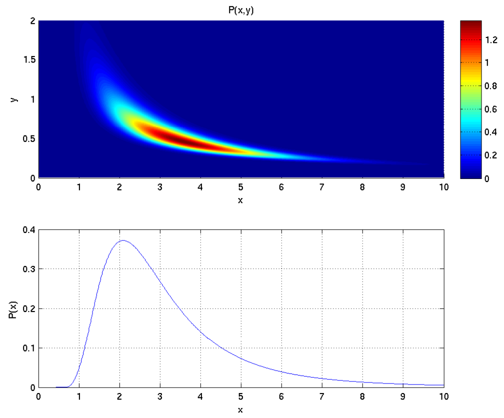

Figure 1: A probability distribution in two variables (x, y), with the marginal distribution in x shown underneath, to illustrate that the projection of the mode of the joint distribution, at 3.55, is in a different place from the mode of the marginal distribution at 2.09.

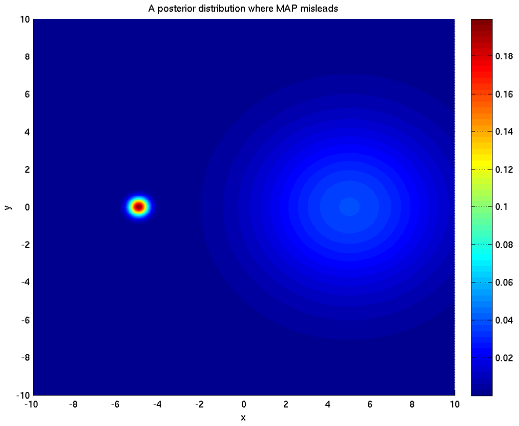

Figure 2: A probability distribution in two variables (x, y) which is bimodal. The MAP value of (x, y) is $(-5, 0)$, but 95% of the probability lies in the broader peak centred at $(+5, 0)$. For another view of this distribution see Figure 3.

(c) Reporting the MAP value is often misleading as far as where the truth is likely to lie — a good example of this is shown in Figure 2, where the MAP value of x is in the middle of the peak centred at $(-5, 0)$, but 95% of the distribution lies in the peak centred at $(+5, 0)$.

For another example, this time from this paper, see section 8.2 of Appendix 2, on testing the software. In this case the MAP often lands not on the correct choice of distribution family, but on one that is under the particular circumstances (parameters) in hand and for the purposes in hand not importantly different from the correct answer; the correct subsequently deduced answer to the problem in hand is correct in all the cases tested.

Nonetheless it is occasionally a reasonable second-best to use MAP, and we have done in this paper for the specific purposes of commenting on software testing and of dithering coincident data values.

2. Various distributions have parameters whose symbols are the same as those in other distributions where they happen to denote specific attributes of the distribution. For example for a Gaussian, μ is both the mean and the median. But for the log-Gaussian, in the parameterisation used here, μ is the log of the median but is *not* the log of the mean.

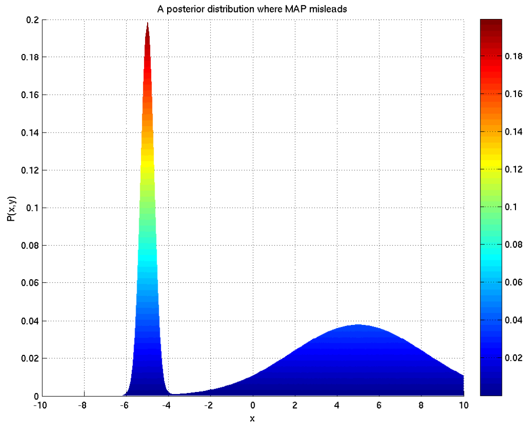

Figure 3: The probability distribution of Figure 2 seen from the direction of $(0, -\infty)$. The MAP value of (x, y) is $(-5, 0)$, but 95% of the probability lies in the broader peak centred at $(+5, 0)$, which is not only much wider than the left-hand peak in the x-direction (as shown) but is also much wider in the y-direction which cannot be seen in this view.

Appendix 2
Supplementary Methods

Contents

1 Introduction — The two problems discussed **2**

2 Overview of the distribution comparison problem **2**

3 Distributions considered **3**

4 Priors on the distribution families **5**

5 Priors on the parameters of each distribution family **5**

5.1 Reference point and units . . . 5
5.2 Gaussian . . . 5
5.3 Log-Gaussian . . . 6
5.4 Student . . . 6
5.5 Log-Student . . . 8
5.6 Gamma . . . 12
5.7 Inverse Gamma . . . 14
5.8 Skew Student . . . 14
5.9 Log-skew-Student . . . 18
5.10 Gamma power . . . 18
5.11 Resulting priors on distributions . . . 22

6 Choice of Q - the restricted geometric mean **22**

7 Carrying out the calculations **23**

8 Testing the software **23**

8.1 Adequacy of grid resolution . . . 23
8.2 Inference of distribution family and direction of comparison . . . 24

9 Comments on necessary practical details **27**

9.1 Calculation in the log domain 27

9.2 Running time and memory requirements 27

9.3 Dealing with data at the extreme bounds of the experimental measurement range 28

10 The probability comparison problem **28**

10.1 Introduction 28

10.2 The questions asked 29

10.3 Answering the first question 29

10.4 Answering the second question 30

11 Provision of software **31**

12 References **31**

1 Introduction — The two problems discussed

The majority of this description of methods focuses on the question of how to work out whether, out of two sets of values, one is bigger than the other — the distribution comparison problem.

However at the end a final section discusses applying similar methods to work out whether, of two probabilities, one is bigger than the other — the probability comparison problem.

Readers who have not encountered Bayesian methods before, and Bayesian model choice in particular, may like to read an informal intuitive introduction in Appendix 1.

2 Overview of the distribution comparison problem

We adopt the Bayesian paradigm. We suppose that we are given two sets of observations A and B of real numbers both distributed according to one of a set F of possible distribution families but with different parameter values. Let $Q(A)$ (resp. $Q(B)$) denote some real scalar property of the distribution from which A (resp. B) is drawn (e.g. arithmetic or geometric mean, median, etc.). We address the general question "What is the probability that $Q(A) > Q(B)$ given A and B ?". We assume in advance that the posterior probability that $Q(A) = Q(B)$ exactly is zero, so the alternative hypothesis being considered is that $Q(A) < Q(B)$.

This problem can be divided into two parts:

1. First we estimate the probabilities, given the data, that each of the distribution families $f \in F$ gives rise to the data. Mathematically, we calculate

$$P(f|A,B) = \frac{P(f)P(A|f)P(B|f)}{\sum_{f \in F} P(f)P(A|f)P(B|f)}.$$

   This involves quantities such as $P(A|f)$, which we calculate by integrating over all possible parameter choices for this family, weighted by their performance on the observed data:

$$P(A|f) = \int P(A|\theta_f, f)P(\theta_f|f)\, d\theta_f,$$

where θ_f is the vector of parameters required by distribution family f.

2. Second, we calculate the probability given the data that $Q(A) > Q(B)$, by

$$P(Q(A) > Q(B)|A,B) = \sum_{f \in F} P(f|A,B) P(Q(A) > Q(B)|f,A,B).$$

In words, we sum the fraction of our hypothesis space, now weighted by evidence, in which $Q(A) > Q(B)$.

In order to do this, we also need to be able to calculate $P(Q(A) > Q(B)|f,A,B)$ which is given by

$$P(Q(A) > Q(B)|f,A,B) = \int_{Q(f,\theta_{f,A}) > Q(f,\theta_{f,B})} P(\theta_{f,A}|f,A) P(\theta_{f,B}|f,B)\, d(\theta_{f,A},\theta_{f,B}),$$

where $Q(f,\theta_f)$ denotes the relevant property of the distribution family f with parameter values θ_f.

The remaining ingredients for these calculations are the prior probabilities $P(f)$ and $P(\theta_f|f)$ for each $f \in F$. These will be discussed in sections 4 and 5 below.

Finally, we report a "significant" result if either $P(Q(A) > Q(B)|f,A,B) > 0.95$ or $P(Q(A) > Q(B)|f,A,B) < 0.05$.

All these methods are standard and long-established; see e.g. [1] chapter 28 or [2].

3 Distributions considered

Based on previous experience with a variety of real world distributions, we chose several reasonable possibilities, and some that can be considered negative controls — the Gaussian, Student, and skew-Student — which fail to exhibit vital characteristics of the data, (e.g., Gaussian and Student distributions are symmetric and put non-zero probability on negative values) and should be rejected if the software is correct. In particular we include the Gaussian because it is a common (but usually false) assumption. The reader should note that these are a set of reasonable possibilities; it is not practical to consider the infinitely many different families of continuous distributions on the positive reals that exist.

When discussing the following distributions the reader may be tempted to assume that e.g. μ denotes the arithmetic mean of the distribution. Although this is true for some of them (e.g. the Gaussian, or the Student when the shape parameter $m > \frac{1}{2}$), in general it is not the case: e.g. the Student has no mean when $m \leq \frac{1}{2}$, and for the log-Gaussian μ is the log of the geometric mean.

Accordingly we define a set F of possible distributions to be the set of the following families, where in each case the vector of parameters following the $|$ on the left-hand side forms the vector θ_f whose posterior distribution will be inferred as part of the procedure (for definitions of symbols not appearing on the LHS of the definitions see after the whole list):

- The Gaussian distribution $P(x|\mu,s) = \sqrt{\frac{s}{2\pi}} e^{-\frac{1}{2}sy^2}$ in which μ is the mean and median and s is the scale, i.e. the reciprocal of the variance;
- The Student distribution $P(x|\mu,s,m) = \frac{1}{\sqrt{2\pi}} \frac{\Gamma(m+\frac{1}{2})}{\Gamma(m)} \frac{r^m}{(r+\frac{1}{2}y^2)^{m+\frac{1}{2}}}$ in which μ is the median, s is the scale (but not necessarily the reciprocal of the variance), and m is a shape parameter which reduces the heaviness of the tails as it gets bigger;
- The log-Gaussian distribution $P(x|\mu,s) = \sqrt{\frac{s}{2\pi}} \frac{1}{x} e^{-\frac{1}{2}sz^2}$ in which μ is the log of the geometric mean and s is the scale, now the reciprocal of the variance of the log value;

- The log-Student distribution $P(x|\mu, s, m) = \frac{1}{\sqrt{2\pi}} \frac{\Gamma(m+\frac{1}{2})}{x\Gamma(m)} \frac{r^m}{(r+\frac{1}{2}z^2)^{m+\frac{1}{2}}}$ in which μ is the log of the median, s is the scale (don't assume anything further), and m is a shape parameter;
- The Gamma distribution $P(x|m, s) = \frac{r^m}{\Gamma(m)} x^{m-1} e^{-rx}$ where m is a shape parameter which makes the distribution narrower as it gets bigger and s is the scale;
- The inverse-Gamma distribution $P(x|m, s) = \frac{r^m}{\Gamma(m)} x^{-m-1} e^{-r/x}$, where the reciprocal of the value is Gamma-distributed;
- The skew-Student distribution (also known as the non-central Student t distribution) [3]

  $$P(x|\xi, m, s, \eta) = \frac{r^m}{\Gamma(m)} \sqrt{\frac{1}{2\pi}} \frac{1}{(r+\frac{1}{2}y^2)^{m+\frac{1}{2}}} e^{-\frac{1}{2}\nu^2} \times \left(\Gamma(m+\frac{1}{2})\,{}_1F_1\left(m+\frac{1}{2}, \frac{1}{2}, \frac{(y\nu)^2}{4(r+\frac{1}{2}y^2)}\right) + \Gamma(m+1) \frac{y\nu}{\sqrt{(r+\frac{1}{2}y^2)}}\,{}_1F_1\left(m+1, \frac{3}{2}, \frac{(y\nu)^2}{4(r+\frac{1}{2}y^2)}\right)\right);$$

  where now ξ is a location parameter, m is a shape parameter (that also affects the skewness), s is a scale parameter, and ν is the skewness parameter;
- The log-skew-Student distribution

  $$P(x|\xi, m, s, \eta) = \frac{r^m}{x\Gamma(m)} \sqrt{\frac{1}{2\pi}} \frac{1}{(r+\frac{1}{2}z^2)^{m+\frac{1}{2}}} e^{-\frac{1}{2}\nu^2} \times \left(\Gamma(m+\frac{1}{2})\,{}_1F_1\left(m+\frac{1}{2}, \frac{1}{2}, \frac{(z\nu)^2}{4(r+\frac{1}{2}z^2)}\right) + \Gamma(m+1) \frac{z\nu}{\sqrt{(r+\frac{1}{2}z^2)}}\,{}_1F_1\left(m+1, \frac{3}{2}, \frac{(z\nu)^2}{4(r+\frac{1}{2}z^2)}\right)\right);$$

  where now the log of the value is skew-Student-distributed;
- The Gamma power distribution $P(x|m, s, k) = |k| \frac{r^m}{\Gamma(m)} x^{mk-1} e^{-rx^k}$ where x^k is Gamma-distributed;

where we make the following identifications:

- $r = ms^k$ (with k taken to be 1 if not applicable, i.e. for all but the Gamma power distribution);
- $y = x - \mu$;
- $z = \log(x) - \mu$;
- $\mu = \xi - \frac{\Gamma(m-\frac{1}{2})}{\Gamma(m)} \nu \sqrt{r}$ for the cases of the (log-)skew-Student only;
- $\nu = \frac{\eta^3 + \alpha\eta}{\eta^2 + \beta}$;
- $\alpha = 100, \beta = 1000$;.

and where Γ denotes the Gamma function defined by $\Gamma(m) = \int_0^\infty x^{m-1} e^{-x}\, dx$, and where ${}_1F_1$ is the hypergeometric function defined by

$${}_1F_1(a, b, z) = \sum_{n=0}^{\infty} \frac{\Gamma(a+n)\Gamma(b) z^n}{\Gamma(a)\Gamma(b+n) n!}.$$

In the case that the data are inherently all positive, as in the present application, those distributions whose support includes the negative reals (i.e. the Gaussian, Student, and skew-Student) should be seen as "straw men"; they cannot possibly be the correct distributions of the data. However, we include them

both because Gaussianity is a common (but usually false) assumption, and in order to demonstrate the ability of the Bayesian approach to reject these possibilities.

We note that all these distributions have several possible ways of parameterising them; e.g. the Gaussian can be parameterised by μ, σ where σ is the standard deviation instead of by μ, s where s is the reciprocal of the variance. Unfortunately which parameterisation is used *does* make a difference to grid resolution needed (and also to speed of convergence of MCMC algorithms) even though it doesn't make any difference to the underlying nature of the distributions; the parameterisations shown are those we recommend.

4 Priors on the distribution families

We consider two priors on the distribution families:

1. $P(f) = \frac{1}{9}$ for all $f \in F$ (i.e. each distribution equally likely).
2. $P(f) = \begin{cases} 1 & (f \text{ is the log-skew-Student family}) \\ 0 & (\text{otherwise}). \end{cases}$, i.e. only the log-skew-Student is considered.

It will later turn out that the conclusions are the same for both priors, even though they are very different. In other words, the log-skew-Student family turns out to be so much more likely (given the data) than the others that any reasonable prior doesn't make any difference.

5 Priors on the parameters of each distribution family

5.1 Reference point and units

We arbitrarily use the geometric mean of the data values for a particular cytokine over all subsets being compared as an absolute scaling factor. In the following that is assumed to be 1.

Logarithms are natural logarithms, i.e. to base e.

In general the choice of parameters for the priors (also known as hyperparameters) was made knowing the basics about the data, namely that it was all positive, but with a wide dynamic range of about 10^5-fold within each cytokine, and with some asymmetry even in the log domain. Beyond that the choices were made to allow a very wide range of distributions for the data to be possible.

5.2 Gaussian

We use the joint conjugate prior to (μ, s) given by

$$P(s|m_s, r_s) = \frac{r_s^{m_s}}{\Gamma(m_s)} s^{m_s-1} e^{-r_s s}[s > 0],$$

$$P(\mu|s, K_\mu, \mu_\mu) = \sqrt{\frac{K_\mu s}{2\pi}} e^{-\frac{1}{2}K_\mu s(\mu-\mu_\mu)^2},$$

where $r_s = m_s s_s$, and set the parameters to $m_s = 4, s_s = 400, K_\mu = 0.2, \mu_\mu = 150$.

This results in the joint distribution for (μ, s) shown in Figure 1, while samples from this family are then shown in Figure 2 on a linear axis and Figure 3 on a logarithmic axis. Note that the prior has been chosen so that there is only a very small chance of distributions allocating more than a few percent of the total probability to the negative reals; this necessarily results in the centre of the distribution family

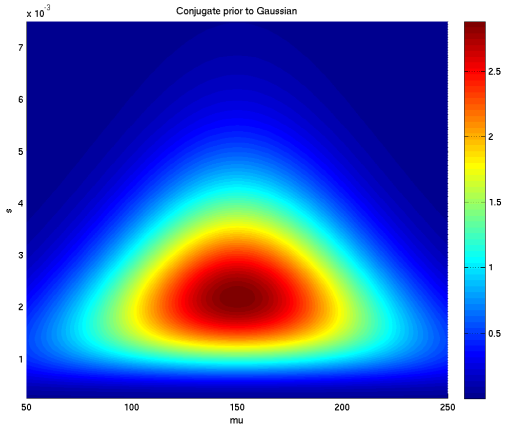

Figure 1: Prior distribution adopted for the parameters μ and s of the Gaussian distribution family

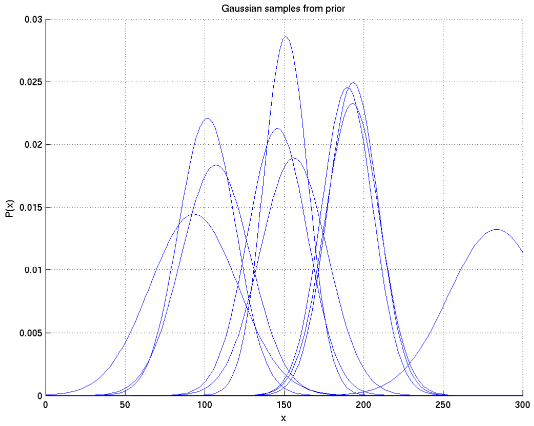

Figure 2: Samples of Gaussian distributions drawn from the prior

being much more positive than we expect the geometric mean of our real life data to be, but to be able to cover the larger samples (which range up to several hundred times the geometric mean).

If we had allowed a substantial fraction of these distributions to be on the negative reals (e.g. if all had 3% there), then we would be penalising the Gaussian family by a factor of $0.97^{250} = 5 \times 10^{-4}$ relative to positive-support families, so Gaussian would get very low posterior probability in step 1 of section 2 above, and there would be even less point including it.

5.3 Log-Gaussian

We adopt the same prior family as for the Gaussian in the previous section, but now with the parameters $m_s = 1.5, s_s = 0.4, K_\mu = 0.2, \mu_\mu = 0$. This results in the joint distribution for (μ, s) shown in Figure 4, while samples from the family are then shown in Figures 5 and 6.

5.4 Student

In this case we use independent priors on each of the parameters μ, m, s. For μ we use a Gaussian with parameters μ_μ, s_μ (i.e. mean μ_μ, variance $1/s_\mu$). For m we use the prior conjugate to the Gamma with

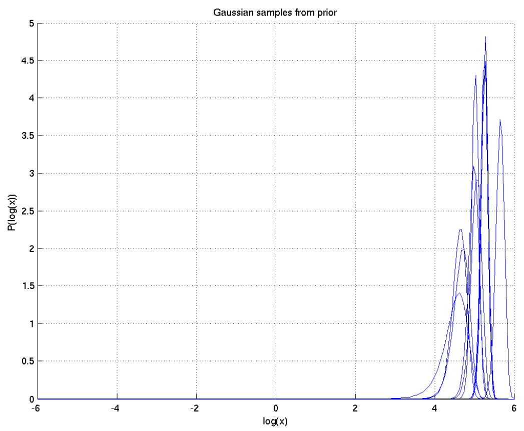

Figure 3: Samples of Gaussian distributions drawn from the prior

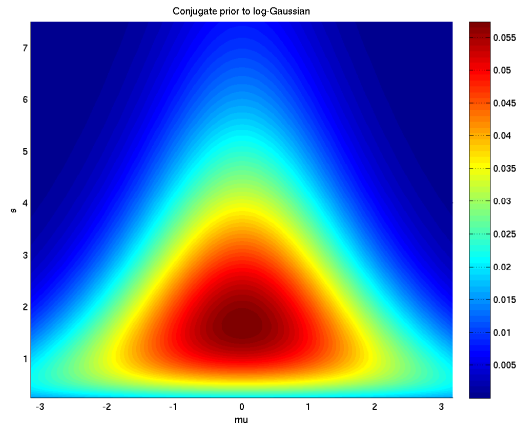

Figure 4: Prior distribution adopted for the parameters μ and s of the log-Gaussian distribution family

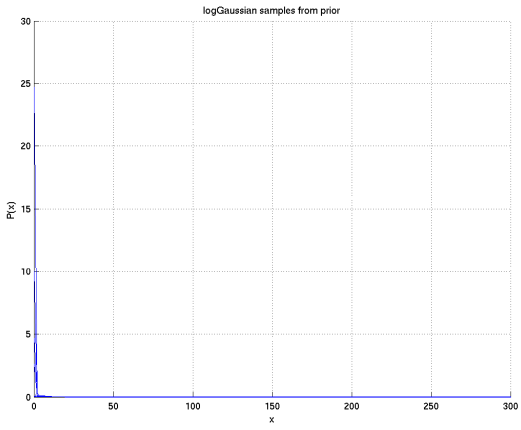

Figure 5: Samples of log-Gaussian distributions drawn from the prior

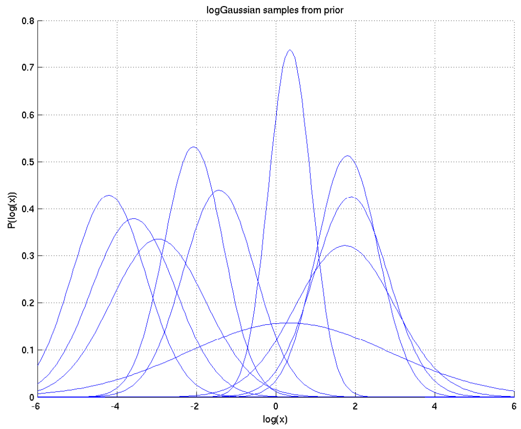

Figure 6: Samples of log-Gaussian distributions drawn from the prior

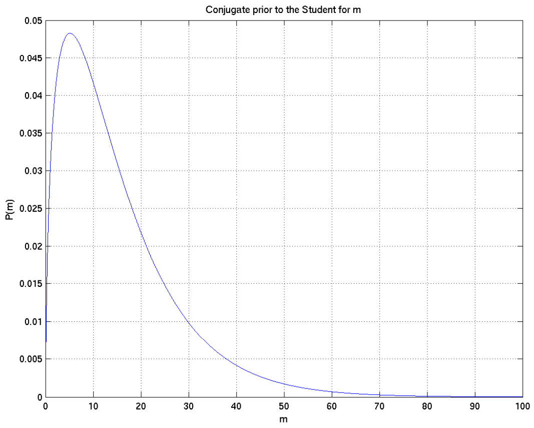

Figure 7: Prior distribution adopted for the parameter m of the Student distribution family

respect to the parameters m, s, namely

$$P(m|a_m, b_m) \propto \frac{e^{-m(a_m+b_m)} m^{b_m m}}{\Gamma(m)^{b_m}} [m > 0],$$

where $a_m > 0, b_m > -1$. For s we use a Gamma prior

$$P(s|m_s, s_s) = \frac{r_s^{m_s}}{\Gamma(m_s)} s^{m_s-1} e^{-r_s s} [s > 0],$$

where $r_s = m_s s_s$, and both r_s and s_s are positive. We set the parameters to $a_m = 0.1, b_m = 1, \mu_\mu = 150, s_\mu = 0.000625, m_s = 1.1, s_s = 0.0025$.

This gives priors on μ, m, s shown in Figures 9, 7, 8 and samples shown in Figures 10 and 11.

5.5 Log-Student

We adopt the same prior families as for the Student above, but with the following parameters: $a_m = 0.2, b_m = 4, \mu_\mu = 0, s_\mu = 1, m_s = 1.1, s_s = 1$. This results in prior distributions on the parameters shown in Figures 12, 13, 14 and samples shown in Figures 15 and 16.

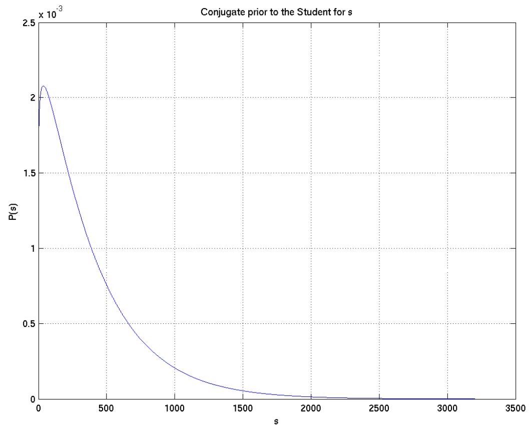

Figure 8: Prior distribution adopted for the parameter s of the Student distribution family

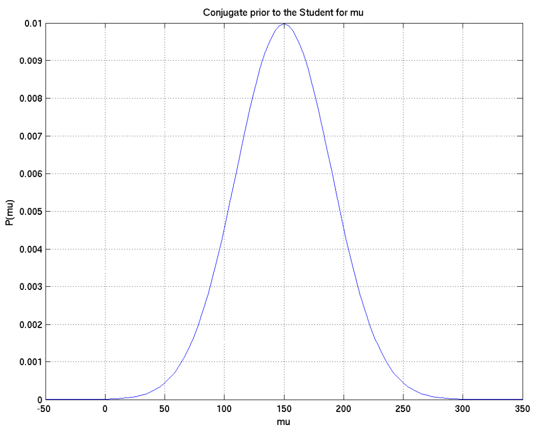

Figure 9: Prior distribution adopted for the parameter μ of the Student distribution family

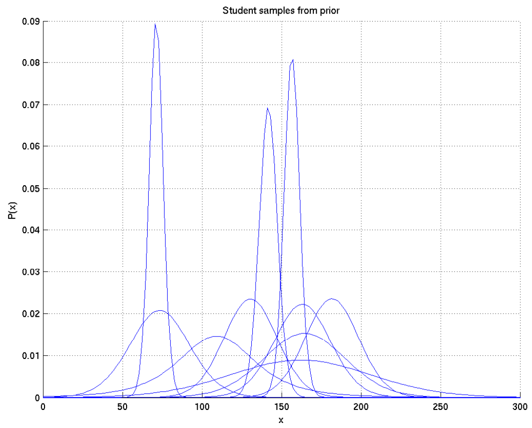

Figure 10: Samples of Student distributions drawn from the prior

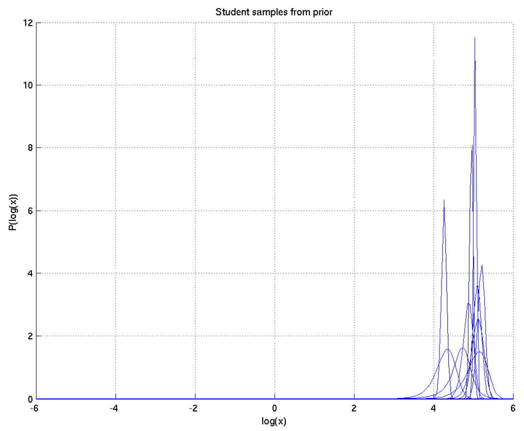

Figure 11: Samples of Student distributions drawn from the prior

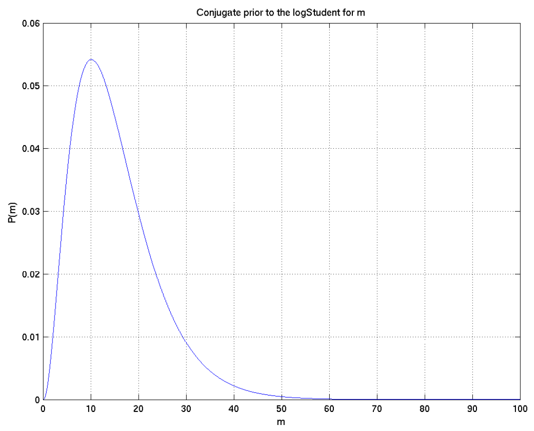

Figure 12: Prior distribution adopted for the parameter m of the log-Student distribution family

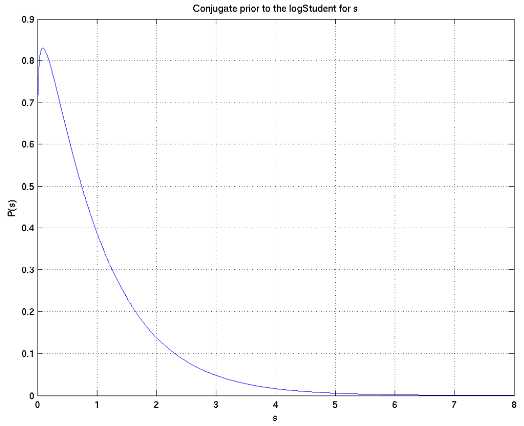

Figure 13: Prior distribution adopted for the parameter s of the log-Student distribution family

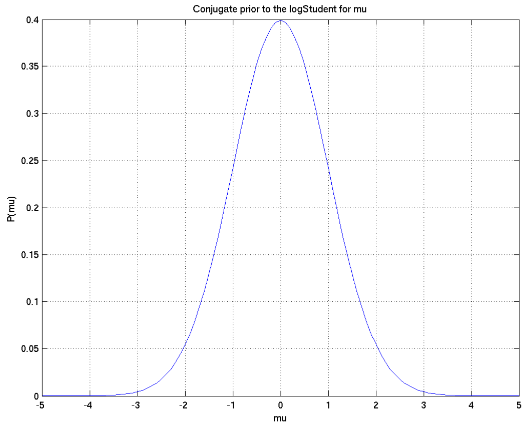

Figure 14: Prior distribution adopted for the parameter μ of the log-Student distribution family

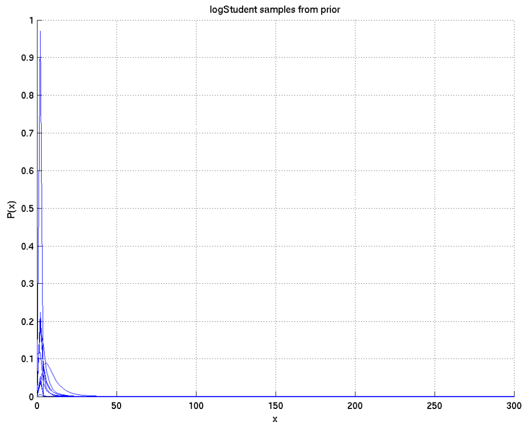

Figure 15: Samples of log-Student distributions drawn from the prior

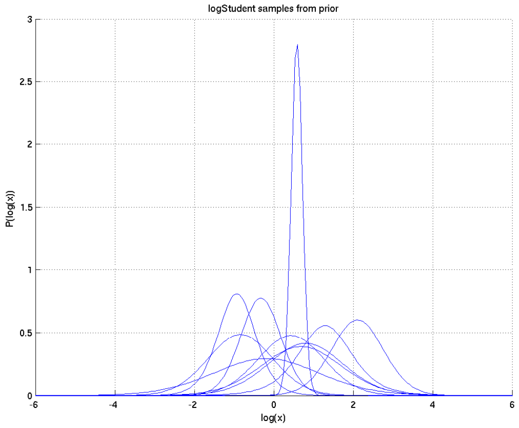

Figure 16: Samples of log-Student distributions drawn from the prior

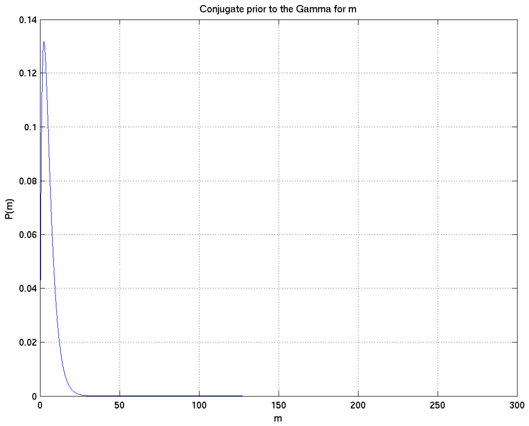

Figure 17: Prior distribution adopted for the parameter m of the Gamma distribution family

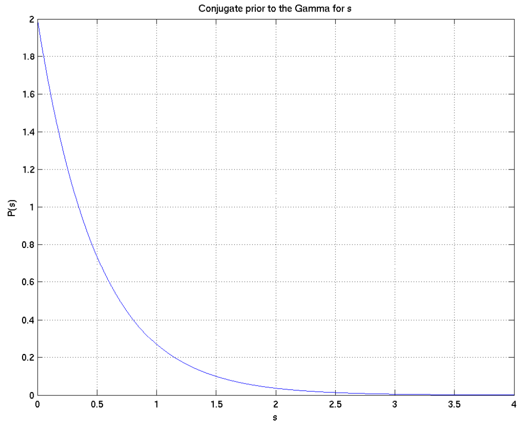

Figure 18: Prior distribution adopted for the parameter s of the Gamma distribution family

5.6 Gamma

We again adopt independent priors for the two parameters. For m we use the prior conjugate to the Gamma with respect to the parameters m, s, namely

$$P(m|a_m, b_m) \propto \frac{e^{-m(a_m+b_m)} m^{b_m m}}{\Gamma(m)^{b_m}} [m > 0],$$

where $a_m > 0, b_m > -1$. For s we use a Gamma prior

$$P(s|m_s, s_s) = \frac{r_s^{m_s}}{\Gamma(m_s)} s^{m_s - 1} e^{-r_s s} [s > 0],$$

where $r_s = m_s s_s$, and both r_s and s_s are positive. We set the parameters to $a_m = 0.02, b_m = 1.5, m_s = 1, s_s = 2$, which results in the prior distributions shown in Figures 17 and 18 and the samples shown in Figures 19 and 20.

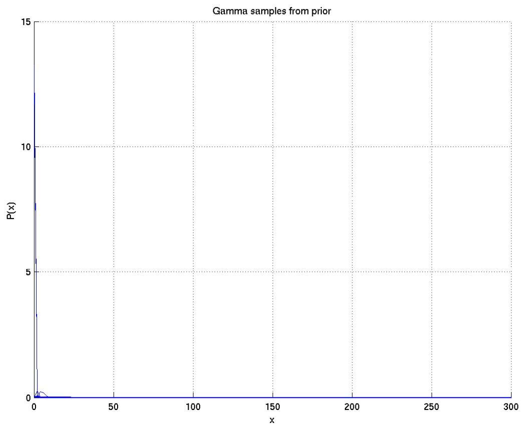

Figure 19: Samples of Gamma distributions drawn from the prior

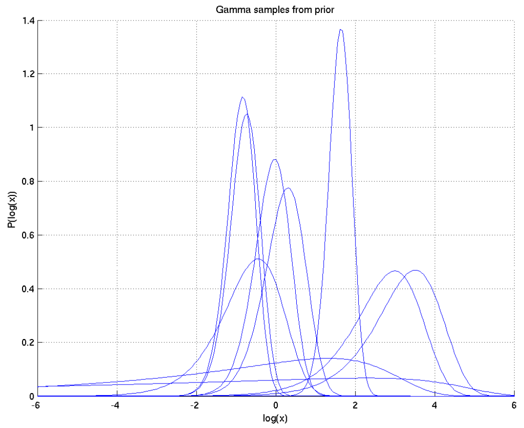

Figure 20: Samples of Gamma distributions drawn from the prior

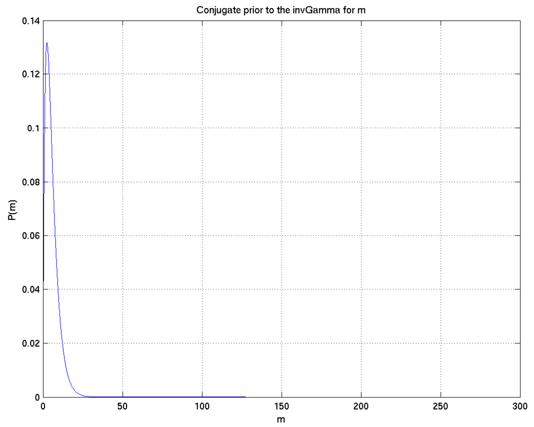

Figure 21: Prior distribution adopted for the parameter m of the inverse-Gamma distribution family

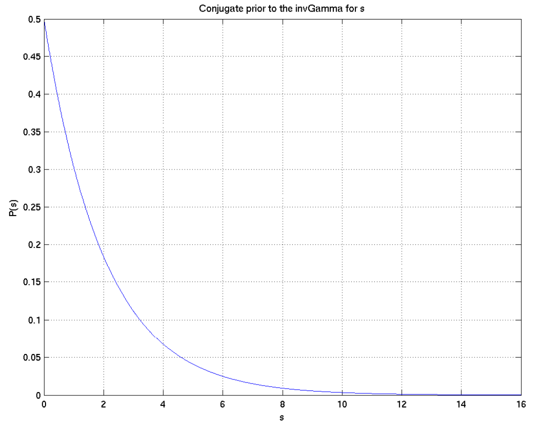

Figure 22: Prior distribution adopted for the parameter s of the inverse-Gamma distribution family

5.7 Inverse Gamma

We use the same priors as for the Gamma distribution, but with the parameters set to $a_m = 0.02, b_m = 1.5, m_s = 1, s_s = 0.5$. This results in the priors on the parameters shown in Figures 21 and 22 and the samples shown in Figures 23 and 24.

5.8 Skew Student

The parameter s has the same prior families as the Student, while ξ is Gaussian with mean μ_ξ and scale s_ξ. We give m the prior family

$$P(m|a_m, b_m) \propto \frac{e^{-m(a_m+b_m)} m^{b_m m}}{\Gamma(m)^{b_m}} [m > \frac{1}{2}].$$

We give η the distribution

$$P(\eta|s_\nu, \alpha, \beta) = \sqrt{\frac{s_\nu}{2\pi}} e^{-\frac{1}{2} s_\nu \left(\frac{\eta^3+\alpha\eta}{\eta^2+\beta}\right)^2} \frac{\eta^4 + (3\beta - \alpha)\eta^2 + \alpha\beta}{(\eta^2 + \beta)^2},$$

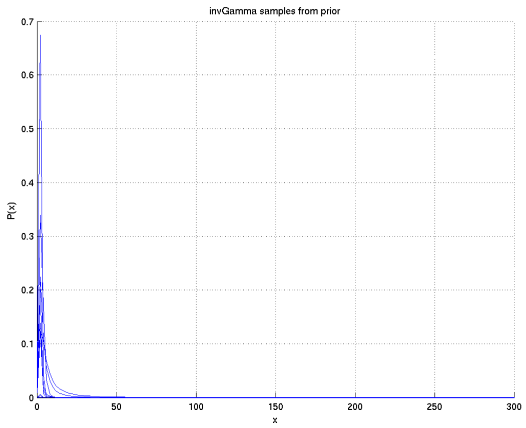

Figure 23: Samples of inverse-Gamma distributions drawn from the prior

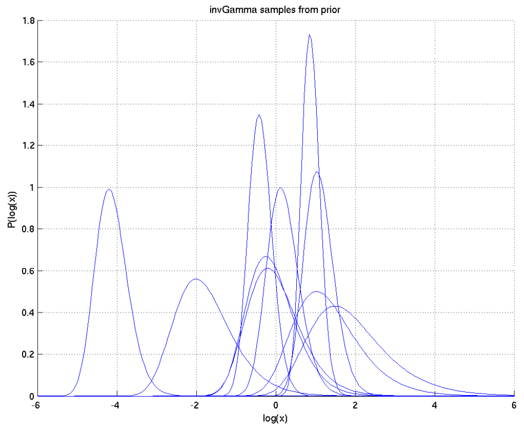

Figure 24: Samples of inverse-Gamma distributions drawn from the prior

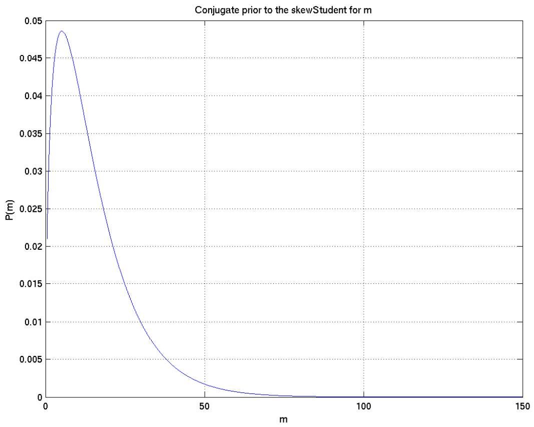

Figure 25: Prior distribution adopted for the parameter m of the skew-Student distribution family

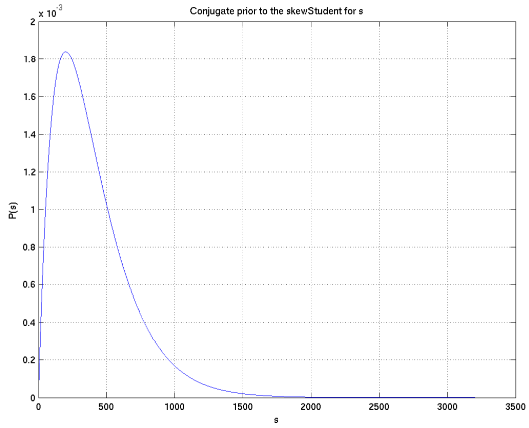

Figure 26: Prior distribution adopted for the parameter s of the skew-Student distribution family

which amounts to making ν Gaussian with zero mean and scale s_ν. The parameters α and β have no effect on the prior distribution of ν, but instead adjust the parameterisation to enable easier numerical integration by making the posterior density smoother, while not changing the posterior distribution.

We adopt the parameters $\alpha = 100, \beta = 1000, s_\nu = 0.25, a_m = 0.1, b_m = 1, m_s = 2, s_s = 0.0025, \mu_\xi = 150, s_\xi = 0.000625$. This results in the prior distributions shown in Figures 25, 26, 27, and 28, and the samples shown in Figures 29 and 30.

Finally we note that in all the final runs for submitted version of the paper the (non-log) skew-Student distribution was omitted from the list of possibilities examined; this was because

- including it nearly doubled the running time (as almost all the running time was spent on the skew-Student and log-skew-Student) and
- preliminary runs had shown that it was not a serious contender to explain these obviously logarithmically-distributed datasets.

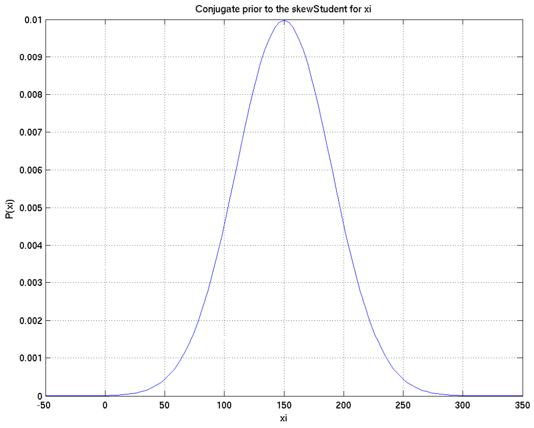

Figure 27: Prior distribution adopted for the parameter ξ of the skew-Student distribution family

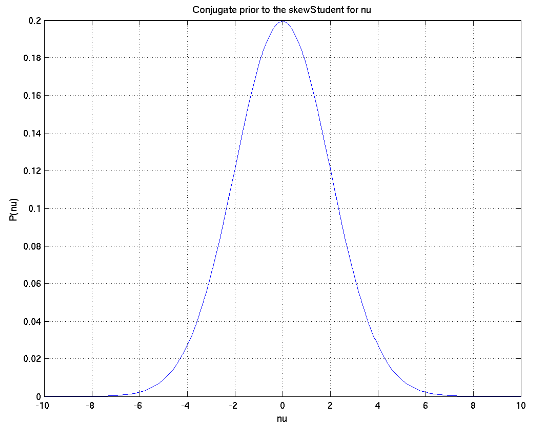

Figure 28: Prior distribution adopted for the parameter ν of the skew-Student distribution family

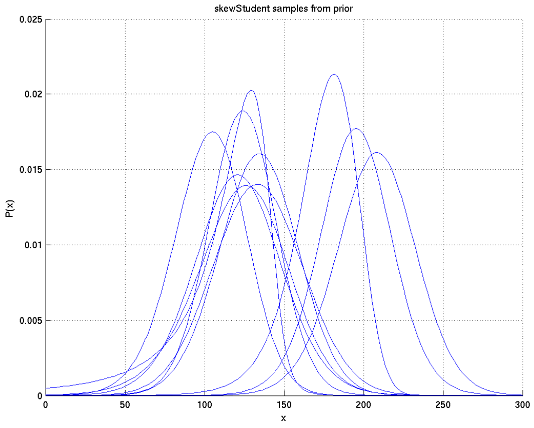

Figure 29: Samples of skew-Student distributions drawn from the prior

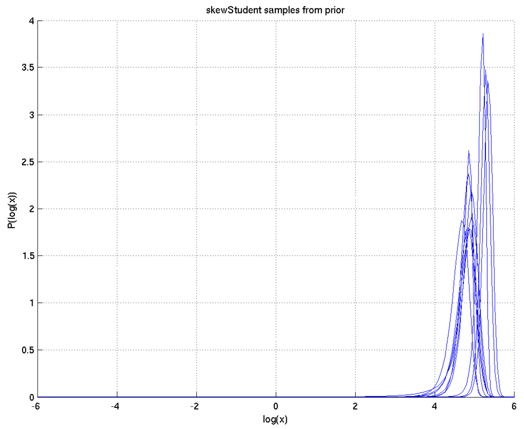

Figure 30: Samples of skew-Student distributions drawn from the prior

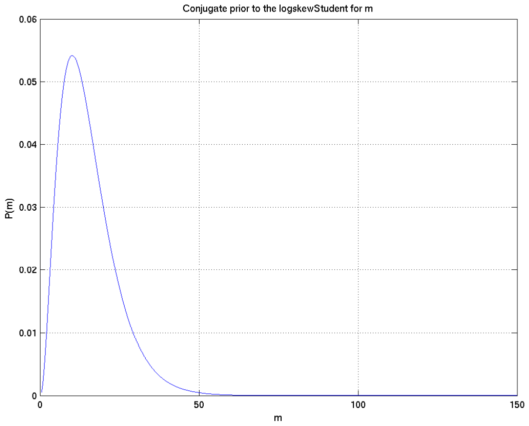

Figure 31: Prior distribution adopted for the parameter m of the log-skew-Student distribution family

5.9 Log-skew-Student

The prior distribution families for the log-skew-Student were chosen to be the same as for the skew-Student, but with the parameters $\alpha = 100, \beta = 1000, s_\nu = 0.025, a_m = 0.2, b_m = 4, m_s = 1.1, s_s = 1, \mu_\xi = 0, s_\xi = 1$. This gives the prior distributions on the parameters shown in Figures 31, 32, 33, and 34, and the samples shown in Figures 35 and 36.

5.10 Gamma power

We take the priors on m and s from the same families with the same parameter values as for the Gamma distribution, except that we set $s_s = 1$.

For k we again choose the conjugate distribution, in this case $P(k|N, a, b, c) \propto k^a \prod_{n=1}^{N} b_n^{kc_n} e^{-c_n b_n^k} [k > 0]$, where N is a positive integer, a a positive scalar, and b, c are N-dimensional vectors of positive reals.

We take $N = 2, a = 1, b = (0.2, 0.2), c = (0.5, 0.5)$, giving the distribution on k shown in Figure 39 and the sample distributions shown in Figures 40 and 41.

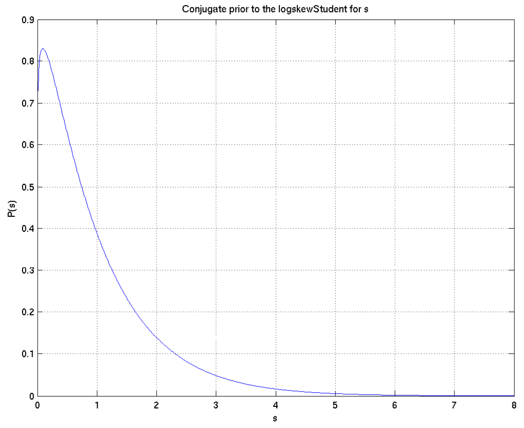

Figure 32: Prior distribution adopted for the parameter s of the log-skew-Student distribution family

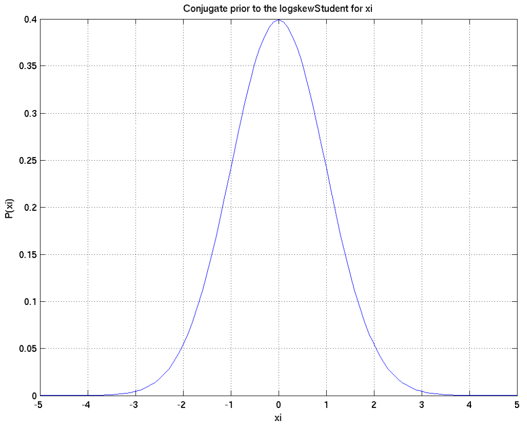

Figure 33: Prior distribution adopted for the parameter ξ of the log-skew-Student distribution family

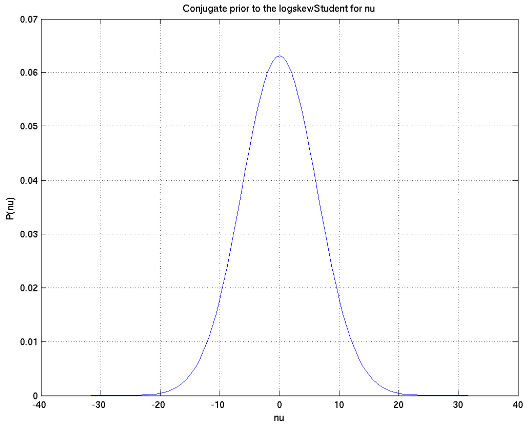

Figure 34: Prior distribution adopted for the parameter ν of the log-skew-Student distribution family

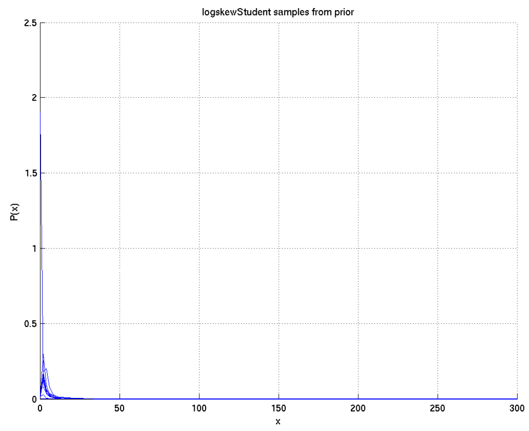

Figure 35: Samples of log-skew-Student distributions drawn from the prior

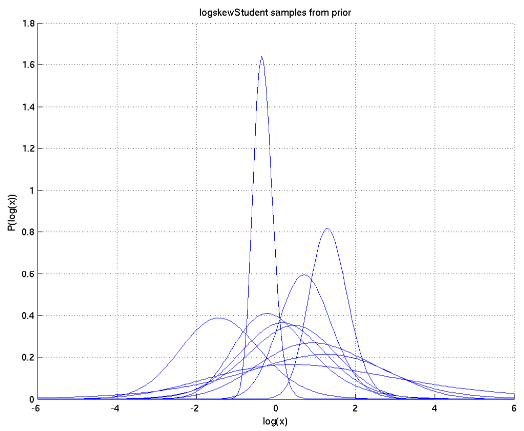

Figure 36: Samples of log-skew-Student distributions drawn from the prior

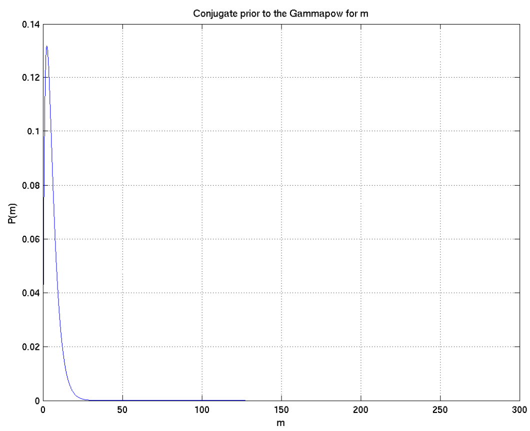

Figure 37: Prior distribution adopted for the parameter m of the Gamma power distribution family

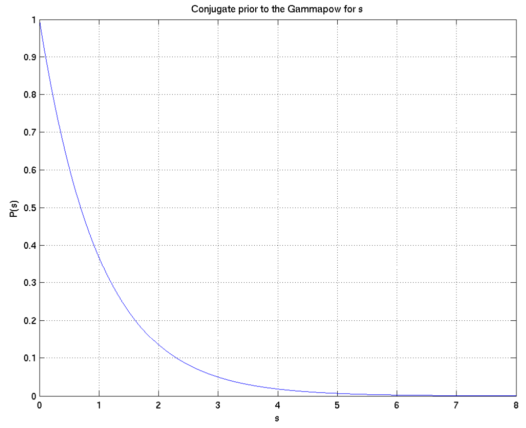

Figure 38: Prior distribution adopted for the parameter s of the Gamma power distribution family

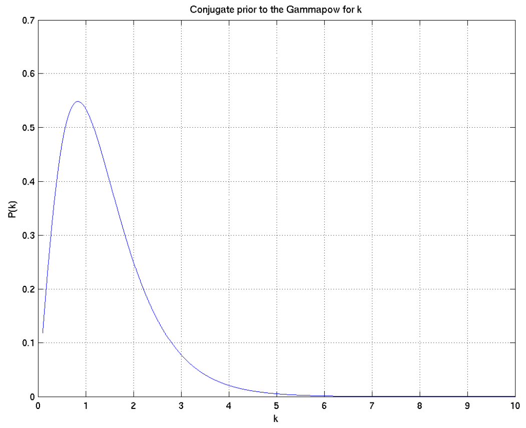

Figure 39: Prior distribution adopted for the parameter k of the Gamma power distribution family

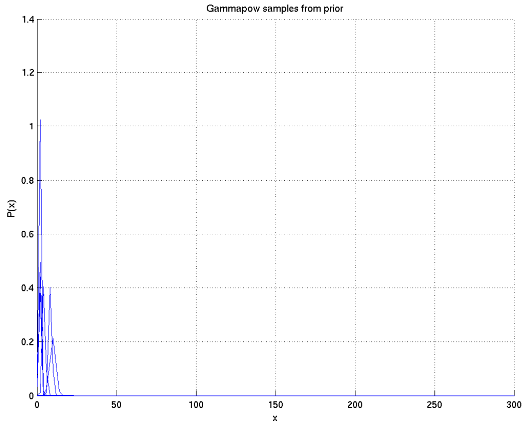

Figure 40: Samples of Gamma power distributions drawn from the prior

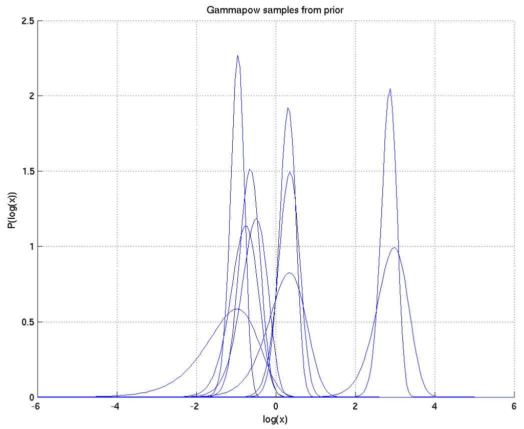

Figure 41: Samples of Gamma power distributions drawn from the prior

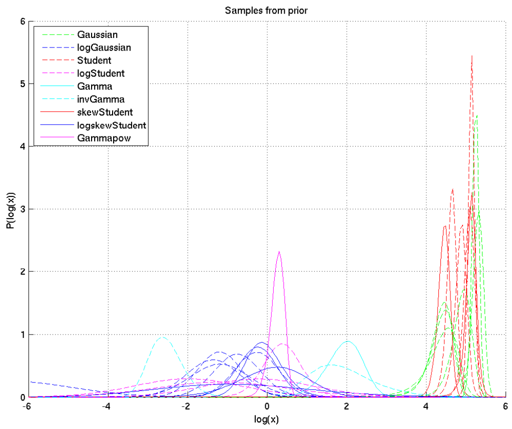

Figure 42: Samples of distributions drawn from the overall prior.

5.11 Resulting priors on distributions

Overall, the priors defined above give us a prior on distributions (as opposed to families) from which samples are illustrated in Figures 42 and 43. For the alternative prior where only log-skew-Students are considered, see Figure 36.

6 Choice of Q - the restricted geometric mean

A further decision that needs to be made is which property of the distribution to use as $Q(f, \theta_f)$. Ordinarily one might expect the mean or median to be used. However, both have drawbacks in this particular situation.

In particular the median will be unaffected by e.g. the top 40% of the distribution's values being doubled, so is insensitive to changes that could be very important.

On the other hand, for five of the distributions being considered, including the one (log-skew-Student) which is will turn out to be by far the most likely single family to give rise to the distribution of all these cytokines, the mean is unfortunately ill-behaved. For the log-(skew)-Student, though the mode is

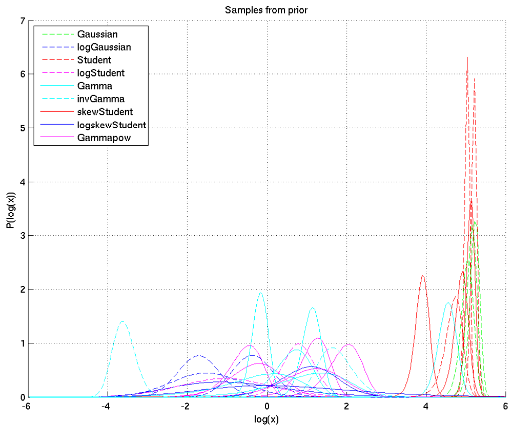

Figure 43: Samples of distributions drawn from the overall prior.

always zero, the mean is always infinite. For the (skew-)Student, the mean is infinite for values of the shape m less than or equal to 0.5, and for the inverse-Gamma it is infinite for values of m less than or equal to 1.

While the geometric mean in some ways behaves better, it does not yet avoid all these problems.

However, by considering instead the restricted geometric mean, i.e. the geometric mean of the part of the distribution between two fixed values $\check{x}$ and $\hat{x}$, the same for all groups being compared, and which are wide enough to encompass e.g. 99% of the probability of all the arising distributions under the posterior and all the observed data values, we obtain a well-behaved and consistent measurement that will capture any likely differences that would be of importance. This we therefore choose as our measurement Q, setting $\check{x} = e^{-6} \approx 0.0025$ times, and $\hat{x} = e^{6} \approx 400$ times the geometric mean of all the observed values of a particular cytokine, which choice turns out to satisfy the above criteria.

7 Carrying out the calculations

In order to carry out the calculations given in section 2 above, we carry out numerical integration on a grid. An automated algorithm was used to determine the grid values in such a way as to include all parts of the distribution with marginal 1-d density in any parameter at least 0.01 times the peak density, and with 40 grid points in each dimension other than that for m for the (log-)skew-Student families, where 20 grid points were used.

The property $Q(f, \theta)$ used was the mean of that part of the distribution between e^{-6} and e^{+6} times the geometric mean of the data from all subgroups of the relevant cytokine; this was evaluated on the given grid for θ and a grid of 200 points log-uniformly spaced over this range.

Matlab was used as the main programming language, with C mex code written to speed up the inner loops (specifically the evaluation of the logarithm of the hypergeometric function).

8 Testing the software

8.1 Adequacy of grid resolution

Adequacy of grid resolution was confirmed by visualising the posterior distributions of the parameters for each distribution family and dataset. We illustrate this by showing that for the log-skew-Student

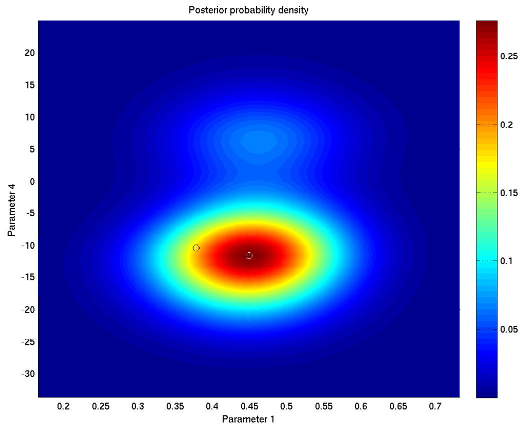

Figure 44: Joint 2-d marginal posterior on parameters ξ and η when inferring parameters of a log-skew-Student model for a synthetic truly log-skew-Student dataset, showing adequacy of grid resolution. The projection of the MAP value of the parameters (ξ, m, s, η) is shown with a white circle and black x and happens to approximately coincide with the mode of this 2-d marginal, while the projection of the true parameter values is shown with a black circle and white x.

inference on a log-skew-Student test dataset in Figure 44. The only times when inadequate gridding was apparent were when the posterior on the (non-log-) skew Student parameters was evaluated for a logarithmically distributed dataset, as for example the data shown in Figures 46 and 47 which gives Figure 45; but it was nonetheless clear that the skew-Student family was very unlikely to be responsible for this dataset as its posterior probability was less than 10^{-48} times that of the log-skew-Student.

8.2 Inference of distribution family and direction of comparison

In order to test the software, we generated two pairs of synthetic datasets from each distribution family, checking if it correctly identified both the family of each pair, and for each pair, which one of the two came from a distribution with higher restricted geometric mean. Each synthetic dataset consisted of 150 points, sampled from the the family, with a sample from the prior for that family determining the parameters.

The posterior probabilities inferred on distribution families, for each synthetic dataset, are shown in Figure 48. Note that we have two pairs for each of 9 families, giving 18 total.

Of the 18 pairs run, by *maximum a posteriori* (MAP) identification (multiple warnings about which are given in Appendix 1):

- one of the two log-Gaussian pairs was identified as log-Student;
- one of the two Student pairs was identified as Gaussian;
- one of the two log-Student pairs was identified as inverse-Gamma;
- one of the two skew-Student pairs was identified as Gaussian;
- one of the two Gamma-power pairs was identified as Gamma.

The remaining 13 pairs of groups were correctly MAP-identified. Of the MAP-misidentified pairs, the lowest probability attached to the correct identification was 0.08; four of the misidentifications were as families with fewer parameters (of which three as subfamilies) and one with more parameters.

As a basic sanity check of the posterior probabilities, we performed the following non-rigorous test:

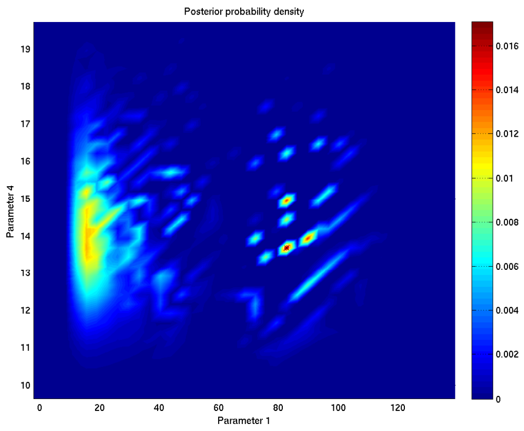

Figure 45: Joint 2-d marginal posterior on parameters ξ and η when inferring parameters of a (non-log-) skew-Student model for a log-skew-Student dataset, showing inadequacy of grid resolution. Nonetheless it was clear that the skew-Student could not account for this distribution, as its posterior probability was less than 10^{-48} times that of the log-skew-Student. The projection of the MAP parameter vector is marked.

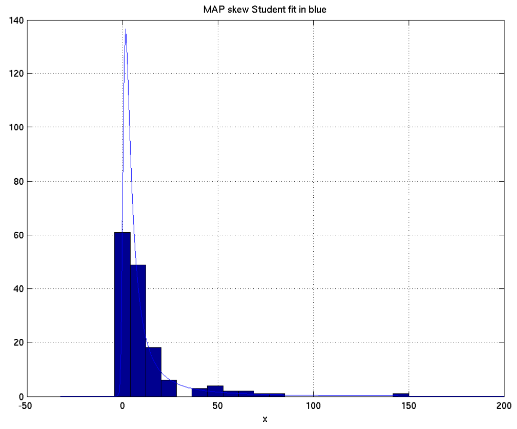

Figure 46: The data that gave rise to Figure 45 and the MAP skew-Student fit on a linear scale; see also Figure 47.

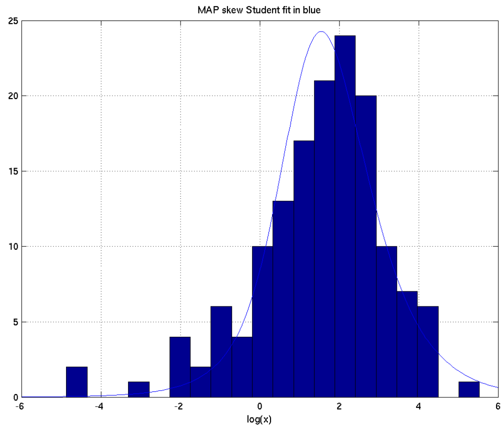

Figure 47: The data that gave rise to Figure 45 and the MAP skew-Student fit on a logarithmic scale, showing how bad the MAP skew-Student fit is to this synthetic log-skew-Student dataset.

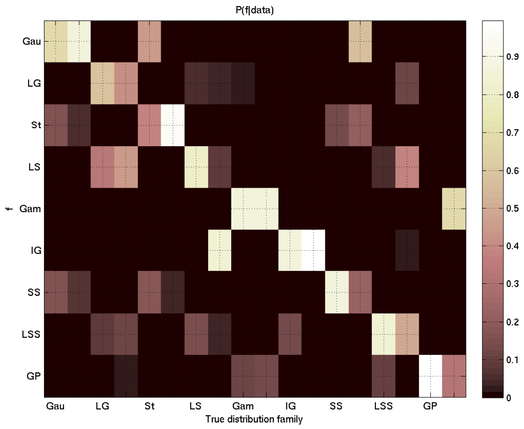

Figure 48: Posterior probabilities of the different possible distribution families for each of the test datasets.

1. Sort the 162 posterior probabilities into order;
2. For each initial segment and final segment of this sequence, count the number that represent the posterior probability of the true family, and apply the (approximate) inverse CDF of the distribution for this that we would expect given the posterior probabilities;
3. Check that all of these quantiles lie between 0.025 and 0.975.

The CDF and thus inverse CDF was approximated by an empirical CDF of 10000 samples from the Bernoulli distribution for each probability.

As seen in Figure 49, which shows these quantiles for each of the 162 possible initial and final segments, we don't observe any outside of our sanity range. If our posterior probabilties had a consistent large bias, or other major problem, we'd expect this simple test to pick up on it.

We observed no bias in favour of distribution families with more parameters, in contrast to maximum likelihood fitting; indeed all but one of the MAP errors observed were in the opposite direction, and all were far from definite misidentifications.

All calculations of $P(Q(f, \theta_{f,A}) > Q(f, \theta_{f,B})|A, B)$ inferred the correct sense of this inequality.

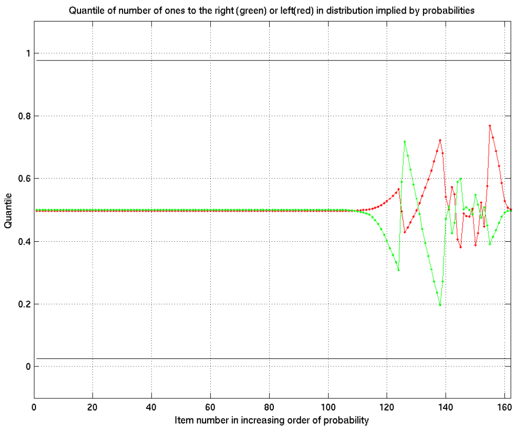

Figure 49: Results of sanity checking of inference software — see text for details.

It is immediately apparent that inference of the family of distributions that generated a particular dataset is based not only on the family definition, but also on the priors on its parameters; e.g. with these priors a dataset all of whose values lie between 100 and 150 is much more likely to be thought to be Gaussian or Student than one whose values all like between 0.001 and 0.1. This is, however, as it should be. As explained e.g. in section 5.2 above, the settings of the parameters are an essential part of practical use of each distribution family.

9 Comments on necessary practical details

In order to carry out these calculations, it is necessary to attend to some critical practical details.

9.1 Calculation in the log domain

The probabilities and densities involved frequently lie outside the range of approximately 10^{-300} to 10^{300} representable in standard floating point arithmetic on a 64-bit computer. In order to ensure that overflow to 0 or ∞ does not occur, it is necessary to work entirely in the log-domain, without moving to the linear domain except for viewing the final answers. Thus e.g. one must use the Matlab `gammaln` function to evaluate $\log(\Gamma(m))$ and not e.g. call `log(gamma(m))`, and similarly for ${}_1F_1$.

9.2 Running time and memory requirements

The implementation used takes approximately 3.5 hours to evaluate a single set of three subgroups of a cytokine on a total of around 400 patients, and requires around 20 Gigabytes of memory in its current implementation. Over 95% of the processing time and memory requirement is used in evaluating the log-skew-Student density, and most of that in evaluating the logarithm of the hypergeometric function ${}_1F_1$. That this is worth-while turns out to be apparent from the calculated posterior probability that these cytokines, if all distributed from the same distribution family, are more than 10^{64} times more likely to be from the log-skew-Student than from any other of the distribution families considered.

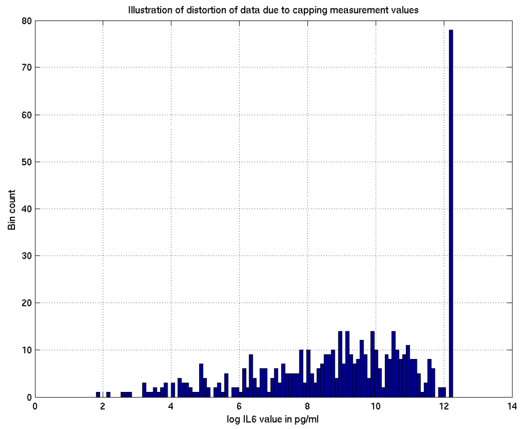

Figure 50: Histogram of samples of IL6, clipped at 210000 pg/ml, distorting the distribution

9.3 Dealing with data at the extreme bounds of the experimental measurement range

Observed distributions can be greatly distorted by forcing all large values to a particular "top of measurement range" value, as illustrated in Figure 50. Similarly this can happen due to finite resolution, and due to clipping values at the bottom of the range.

In order that the results are not distorted by this, we did the following steps, for which process we use the common term "dithering":

1. We first inferred the distribution types as in part 1 of section 2.
2. For each value to be replaced, we:
    (a) Drew a family at random with probability equal to the posterior probability of that family;
    (b) Used the MAP sample of the parameters of that distribution to give a specific distribution from that family;
    (c) Replaced the clipped value with a random sample from that distribution, conditional on being within the range possible given the finite resolution and any clipping and the observed value.

Such random sampling was done by rejection sampling, easy to program though not efficient. We then reran the entire analysis using as input data the resampled clipped values.

(Note that we do not recommend MAP sampling in general; this was merely a quick and dirty expedient which was cheap in compute power and programming effort to execute. In an ideal world we would recommend Markov-chain Monte Carlo to infer the unclipped values, but in this context the workload would be too great. Indeed if we had used a random posterior sample of the parameters rather than the MAP value what we did would be precisely the first step of such an MCMC process.)

10 The probability comparison problem

10.1 Introduction

We now turn to a different question, namely assessment of whether a decision to collect cytokine measurements by the clinicians is influenced by the values of certain other variables. In particular we take

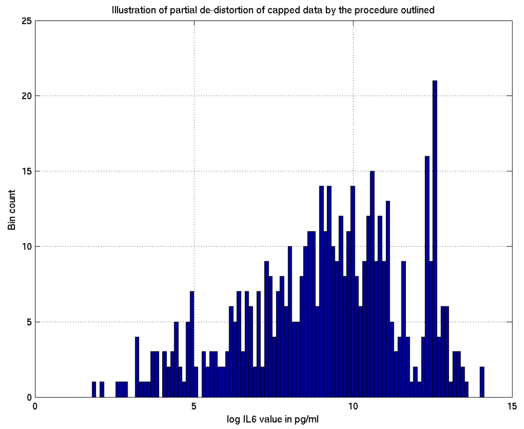

Figure 51: Histogram of samples of IL6, after resampling the clipped values from the MAP inferred distribution. Not perfect, but a great deal better than the unresampled version.

a subset S of the patients, separate them into those without and with a particular variable (S_0, S_1 respectively), and we consider the respective probabilities p_0 and p_1 that a patient from S_0 or S_1 has their cytokines measured.

As data for this we have the values N_0 and N_1, the total numbers of patients in S_0 and S_1, and n_0 and n_1, the respective numbers who did have their cytokines measured. In the majority of cases a patient either had all or no cytokines measured; for the few for whom this was not true, we took measurement or not of TNFα as the defining datum.

10.2 The questions asked

We then asked two questions:

1. Consider two models for what we think about p_0, p_1 before collecting the data, namely M_1 in which we are 100% sure that $p_0 = p_1$ and that this common value is uniformly distributed on $[0, 1]$, and M_2 in which we believe that p_0 and p_1 are independently uniformly distributed on $[0, 1]$ (so that the probability that they are exactly equal is zero). Now suppose we think the prior probabilities that M_1 holds and that M_2 holds are equal (and are both $\frac{1}{2}$). Now, in the light of the data, how likely is it that M_2 is the correct model ?

2. Suppose now that M_2 is the correct model. How likely is it that $p_0 > p_1$?

10.3 Answering the first question

Similarly to the method of model choice used when considering different distribution families, we proceed as follows.

Applying Bayes' theorem we find

$$P(M_2|n_0, n_1, N_0, N_1) = \frac{P(M_2)P(n_0, n_1|M_2, N_0, N_1)}{\sum_{k=1}^{2} P(M_k)P(n_0, n_1|M_k, N_0, N_1)} = \frac{P(n_0, n_1|M_2, N_0, N_1)}{\sum_{k=1}^{2} P(n_0, n_1|M_k, N_0, N_1)}.$$

But now

$$\begin{aligned}
P(n_0, n_1|M_1, N_0, N_1) &= \int P(n_0, n_1, p|M_1, N_0, N_1)\, dp \\
&= \int P(n_0, n_1|p, M_1, N_0, N_1) P(p|M_1, N_0, N_1)\, dp \\
&= \int P(n_0, n_1|p, M_1, N_0, N_1)\, dp \\
&= \int \frac{N_0!}{n_0!(N_0-n_0)!} p^{n_0}(1-p)^{N_0-n_0} \frac{N_1!}{n_1!(N_1-n_1)!} p^{n_1}(1-p)^{N_1-n_1}\, dp \\
&= \int \frac{N_0! N_1!}{n_0!(N_0-n_0)! n_1!(N_1-n_1)!} p^{n_0+n_1}(1-p)^{N_0-n_0+N_1-n_1}\, dp \\
&= \frac{N_0! N_1!}{n_0!(N_0-n_0)! n_1!(N_1-n_1)!} \frac{\Gamma(n_0+n_1+1)\Gamma(N_0-n_0+N_1-n_1+1)}{\Gamma(N_0+N_1+2)}
\end{aligned}$$

(where $\Gamma(m)$ denotes the Gamma function given by $\Gamma(m) = \int_0^\infty x^{m-1} e^{-x}\, dx$) while

$$\begin{aligned}
P(n_0, n_1|M_2, N_0, N_1) &= \int P(n_0, n_1, p_0, p_1|M_2, N_0, N_1)\, d(p_0, p_1) \\
&= \int P(n_0, p_0|N_0)\, dp_0 \int P(n_1, p_1|N_1)\, dp_1
\end{aligned}$$

and for $k = 0, 1$,

$$\begin{aligned}
\int P(n_k, p_k|N_k)\, dp_k &= \int P(p_k|N_k) P(n_k|p_k, N_k)\, dp_k \\
&= \int P(n_k|p_k, N_k)\, dp_k \\
&= \int \frac{N_k!}{n_k!(N_k-n_k)!} p_k^{n_k}(1-p_k)^{N_k-n_k}\, dp_k \\
&= \frac{N_k!}{n_k!(N_k-n_k)!} \frac{\Gamma(n_k+1)\Gamma(N_k-n_k+1)}{\Gamma(N_k+2)}.
\end{aligned}$$

Putting all this together we find that

$$\begin{aligned}
P(M_2|n_0, n_1, N_0, N_1) &= \frac{\prod_{k=0}^1 \frac{N_k!}{n_k!(N_k-n_k)!} \frac{\Gamma(n_k+1)\Gamma(N_k-n_k+1)}{\Gamma(N_k+2)}}{\prod_{k=0}^1 \frac{N_k!}{n_k!(N_k-n_k)!} \frac{\Gamma(n_k+1)\Gamma(N_k-n_k+1)}{\Gamma(N_k+2)} + \frac{N_0! N_1!}{n_0!(N_0-n_0)! n_1!(N_1-n_1)!} \frac{\Gamma(n_0+n_1+1)\Gamma(N_0-n_0+N_1-n_1+1)}{\Gamma(N_0+N_1+2)}} \\
&= \frac{\prod_{k=0}^1 \frac{\Gamma(n_k+1)\Gamma(N_k-n_k+1)}{\Gamma(N_k+2)}}{\prod_{k=0}^1 \frac{\Gamma(n_k+1)\Gamma(N_k-n_k+1)}{\Gamma(N_k+2)} + \frac{\Gamma(n_0+n_1+1)\Gamma(N_0-n_0+N_1-n_1+1)}{\Gamma(N_0+N_1+2)}}
\end{aligned}$$

which can now be easily evaluated e.g. in Matlab.

10.4 Answering the second question

Here we have

$$\begin{aligned}
P(p_0 > p_1|n_0, n_1, N_0, N_1) &= \int_{\{(p_0,p_1):p_0>p_1\}} P(p_0, p_1|n_0, n_1, N_0, N_1)\, d(p_0, p_1) \\
&= \int_{\{(p_0,p_1):p_0>p_1\}} P(p_0|n_0, N_0) P(p_1|n_1, N_1)\, d(p_0, p_1)
\end{aligned}$$

while for $k = 0, 1$,

$$\begin{aligned} P(p_k|n_k, N_k) &= \frac{P(p_k)P(n_k|p_k, N_k)}{\int_0^1 P(p_k)P(n_k|p_k, N_k)\, dp_k} \\ &= \frac{P(n_k|p_k, N_k)}{\int_0^1 P(n_k|p_k, N_k)\, dp_k} \\ &= \frac{p_k^{n_k}(1-p_k)^{N_k-n_k}}{\int_0^1 p_k^{n_k}(1-p_k)^{N_k-n_k}\, dp_k} \\ &= \frac{\Gamma(N_k+2)}{\Gamma(n_k+1)\Gamma(N_k-n_k+1)} p_k^{n_k}(1-p_k)^{N_k-n_k}, \end{aligned}$$

following which the required integral can be numerically evaluated on a grid.

11 Provision of software

Matlab software for the distribution comparison problem is provided in the supplementary file

“`Dataset S03 (GZ)`”

which is a 64-bit Linux tarball, and should be renamed after downloading e.g. to

`distribution_comparison_software.tar.gz`

and then uncompressed to its individual files using the Linux command

`tar -xzf distribution_comparison_software.tar.gz`

A list of the primary entry points is in the component file `primaryentrypoints`, each of which has Matlab help text at its head.

12 References

References

[1] D. MacKay, *Information Theory, Inference, and Learning Algorithms.* Cambridge University Press, 2003.

[2] Radford M. Neal, “Probabilistic inference using Markov Chain Monte Carlo Methods,” *1993*, Technical Report CRG-TR-93-1, Department of Computer Science, University of Toronto.

[3] K. Levy and S. Narula, “Probability density plots of the non-central t-distribution,” *International Statistics Review*, vol. 42, pp. 305–306, 1974.
